# Pesticide Surveillance in Fruits and Vegetables from Romanian Supply: A Data-Driven Approach

**DOI:** 10.3390/jox15040104

**Published:** 2025-07-02

**Authors:** Diana Ionela Popescu (Stegarus), Ana-Maria Nasture, Violeta-Carolina Niculescu, Corina Mihaela Oprita (Cioara), Nicoleta Anca Șuțan (Ionescu)

**Affiliations:** 1 National Research and Development Institute for Cryogenic and Isotopic Technologies, ICSI Ramnicu Valcea, 4th Uzinei Street, 240050 Ramnicu Valcea, Romania; diana.stegarus@icsi.ro (D.I.P.);; 2 Doctoral School of Applied Sciences, Ovidius University Constanta, 124 Mamaia Blvd, 1st University Alley, 900470 Constanta, Romania; 3 Department of Natural Sciences, National University of Science and Technology POLITEHNICA Bucharest, Pitesti University Centre, Targu din Vale Str. No. 1, 110040 Pitesti, Romania

**Keywords:** pesticides, fruits, vegetables, chromatography, statistical analysis

## Abstract

The evolution of global agriculture encourages the extensive use of pesticides although significant concerns regarding their impact on human health and the environment must be considered. The present paper highlights the presence and concentrations of various pesticide residues in fruits and vegetables available on Romanian markets. A total of 74 pesticide compounds authorized for agricultural use were identified and quantified in 620 randomly selected samples spanning a wide range of horticultural products by employing the QuEChERS extraction method and liquid chromatography–mass spectrometry (LC-MS/MS). The most often detected pesticides comprised boscalid and azoxystrobin, present in 42% and 37% of apple and strawberry samples, respectively, with mean concentrations of 0.12 mg/kg and 0.09 mg/kg. In cucumbers and tomatoes, difenoconazole and acetamiprid were predominant, detected in 35% and 40% of samples, with average residue amounts of 0.08 mg/kg and 0.07 mg/kg, respectively. Statistical analysis, achieved with Python 3.13.2, the pandas library (alongside descriptive statistics), and ANOVA, revealed significant variations in residue levels based on the product type and geographic origin. Boscalid and azoxystrobin were commonly encountered in apples and strawberries while difenoconazole and acetamiprid predominated in cucumbers and tomatoes. Even though the majority of pesticide residues conformed to EU maximum residue limits (MRLs), about 6% of samples, generally from imported products, displayed some residue concentrations approaching critical thresholds, with the highest exceedance observed for chlorpyrifos and lambda-cyhalothrin at concentrations of up to 0.25 mg/kg. This research provides a comprehensive overview of pesticide residues prevalence in Romania’s fresh product supply while, at the same time, supporting consumer awareness initiatives and evidencing the critical demand for continuous monitoring and strengthened regulatory frameworks for food safety.

## 1. Introduction

Global agricultural intensification has driven the development of multiple chemical formulations, referred to as pesticides, aimed at controlling pests and diseases. Pesticides encompass a broad spectrum of chemical substances, including both organic and inorganic compounds, such as insecticides, herbicides, rodenticides, fungicides, nematicides, molluscicides, and plant growth regulators [1,2]. The concept associated with pesticides varies by country but generally refers to a set of compounds that should be toxic to target organisms and harmless to the environment, ecosystems, and humans [3,4,5].

The intensification of global agriculture has relied heavily on synthetic pesticides to meet rising food demands, with the worldwide usage of active ingredients reaching 3.5 million tons in 2021 [6,7] and 3.7 million tons in 2022 according to the Food and Agriculture Organization of the United Nations [8]. While these chemicals mitigate crop losses from pests and diseases, their pervasive application has led to widespread environmental contamination and human health risks [6,9]. The European Union (EU) has established stringent food safety standards under Regulation No. 396/2005, delineating a comprehensive set of requirements and establishing maximum residue limits (MRLs) for over 1300 pesticides [10]. The Codex Alimentarius Commission (CODEX), a collaborative body of the WHO and FAO, maintains a rigorous framework for the establishing of MRLs of pesticides present in food products [11].

However, the globalization of food supply chains introduces risks as imported produce from non-EU countries often originates from regions with laxer pesticide regulations or enforcement. A European Food Safety Authority (EFSA) report found that 6.5% of non-EU fruit imports exceeded EU maximum residue limits (MRLs), compared to 1.7% of intra-EU samples [12]. This disparity highlights vulnerabilities in border surveillance between producer and consumer nations.

Pesticide residues persist in soil, water, and food chains, contributing to bioaccumulation and chronic exposure linked to neurotoxicity, endocrine disruption, and carcinogenicity [13]. Even in regulated markets, residues of systemic insecticides like neonicotinoids and fungicides such as succinate dehydrogenase inhibitors (SDHIs) are routinely detected in staple fruits and vegetables, raising alarms about dietary exposure [12].

Exposure to pesticides that may exhibit cytotoxic, carcinogenic, and mutagenic properties, especially though food-related risks, can lead to chronic or acute health conditions [14]. The action pathways of pesticides extend beyond a single target pest as there may be direct or indirect interactions with other organisms [15,16]. The toxicity level of pesticides is related with the electronic and structural characteristics of the molecules alongside exposure times and dosages used [17,18].

The long-term active presence of pesticides in treated areas has led regulatory authorities of the EU to prohibit the agricultural usage of certain substances. Out of approximately 1300 commercially available active compounds, between 820 and 850 have been prohibited [2]. The extremely high demand for agricultural production and the necessity of ensuring global food supply have created a mandatory condition for pesticide use.

Romania, a major EU agricultural hub and importer of fruits/vegetables from neighboring EU and non-EU states (e.g., Moldova, Turkey, Egypt), faces unique risks. Despite adherence to EU MRLs for domestically grown produce, the surveillance of imported goods remains fragmented. A 2021 study detected unauthorized pesticides in 12% of Romanian market samples, yet comprehensive data on contamination patterns, particularly for multi-residue interactions and non-EU imports, are lacking [19]. This gap undermines consumer safety and complicates policy responses to emerging contaminants. Apart from regular domestic surveillance, targeted research has been achieved on imported fruits and vegetables, mainly on those originating from non-EU countries. For example, sporadic investigations have identified unauthorized or banned pesticides in imported commodities, highlighting potential gaps in border control and import surveillance [20]. Conventional surveillance methods frequently fail to detect the complexity of pesticide mixtures and underestimate the exposure risks to consumers [21]. Some studies highlighted that the integration of advanced multi-residue analytical methods allows the more accurate detection of a broader spectrum of pesticides, including metabolites and degradation products, essential for realistic risk assessments [21]. Moreover, the outcomes have highlighted the demand for harmonized surveillance protocols and improved regulatory oversight, mainly in the context of globalized food supply chains, where imported produce may introduce non-compliant residues [21]. Nevertheless, these efforts remain fragmented and unharmonized, with a limited integration of advanced multi-residue analytical protocols able of simultaneously detecting hundreds of pesticides and their metabolites.

Furthermore, there is also a scarcity of systematic data on pesticide residues originating from imports from a wide range of geographic origins, including non-EU countries, which frequently use various pesticide regulations and standards [22]. This deficiency weakens comprehensive risk assessments and restricts the formulation of efficient, evidence-based regulations to protect consumers from emerging contaminants and multi-residue exposures. Consequently, extending and harmonizing surveillance initiatives, incorporating state-of-the-art multi-residue detection technologies, and strengthening import controls are critical to addressing these challenges and safeguarding public health [19].

The hypothesis of this study was that the pesticide contamination patterns in fruits and vegetables available on the Romanian market may vary significantly, depending on the product type and geographical origin, mainly between EU and non-EU imports. Considering this, the main objectives of the study were to identify and quantify pesticide residues in a wide range of fresh fruits and vegetables sold in Romania using LC-MS/MS analysis, to assess compliance with EU maximum residue limits (MRLs), to analyze statistical differences in pesticide residues based on product type and origin using ANOVA and descriptive statistics, and to identify contamination patterns through K-means clustering and visualize distribution trends using interactive charts. This integrated, data-driven approach aimed to highlight regulatory protocols and raise consumer awareness regarding pesticide residues in the food supply.

## 2. Materials and Methods

In 2023, a total of 620 samples comprising 37 types of fresh fruits and vegetables were collected from Romanian supermarkets. The samples represented imports from Albania, Argentina, Bulgaria, China, Colombia, Costa Rica, Ecuador, Egypt, France, Germany, Greece, Hungary, India, Israel, Italy, Kenya, Macedonia, Moldova, the Netherlands, Poland, Romania, Serbia, South Africa, Spain, Tanzania, Tunisia, Turkey, and Ukraine.

The examined sample set consisted of 84 samples of oranges, 84 samples of lemons, 78 samples of bananas, 47 samples of grapefruits, 42 samples of apples, 41 samples of grapes, 37 samples of mandarins, 24 samples of pomelos, 21 samples of pears, 11 samples of pomegranate, 9 samples of apricot, 8 samples of strawberry, 6 samples of clementine, 5 samples of plum, 5 samples of peaches, 5 samples of quinces, 4 samples of nectarines, 3 samples of watermelons, 1 sample of sour cherries, 1 sample of kiwi, 1 sample of cherry, 30 samples of peppers, 29 samples of tomatoes, 18 samples of cucumbers, 7 samples of zucchini, 6 samples of carrots, 2 samples of eggplants, 2 samples of peas, 1 sample of cabbages, 1 sample of garlic, 1 sample of celery, 1 sample of lettuce, 1 sample of leek, 1 sample of bell pepper, 1 sample of pumpkin, 1 sample of onion, and 1 sample of broccoli.

Each sample, with a weigh of at least 1 kg, was homogenized, sealed in food bags, and stored at −20 °C until processing.

### 2.1. Reagents

The used high-purity reagents (Merck Group, Darmstadt, Germany) included acetonitrile (purity ≥ 99.9%), methanol (purity ≥ 99.9%), ammonium formate (1M), formic acid (purity > 95%), and internal standard triphenyl phosphate (TPP). Ultrapure water used to ensure the quality of the prepared solutions was produced with a Millipore Direct Q 3UV system (MilliporeSigma, part of Merck KGaA, Darmstadt, Germany). The QuEChERS (Quick, Easy, Cheap, Effective, Rugged, Safe) method involved the use of two specific formulations, containing acetonitrile (LC-MS grade), magnesium sulfate (MgSO_4_), sodium chloride (NaCl), sodium citrate tribasic dihydrate, and disodium hydrogen citrate sesquihydrate (C_6_H_5_Na_3_O_7_·2H_2_O) purchased from Merck KGaA (Darmstadt, Germany).

The Quechers (buffer salt mixture, sorbents for dispersive SPE (dispersive solid-phase extraction) including primary secondary amine (PSA) and graphitized carbon black C18 (GCB), and tubes) were purchased from Diamedix Diagnostica (Bucharest, Romania).

### 2.2. Extraction and Cleanup Procedure

All samples were prepared using the modified QuEChERS method [23,24]. A total of 10 g of homogenized sample were weighed into 50 mL centrifuge tube, followed by the addition of 50 µL 10 µg mL^−1^ TPP as internal standard. The tubes were sealed and vigorously shaken. Subsequently, 10 mL acetonitrile was added, and the samples were shaken for 20 min using a Heidolph orbital shaker (Heidolph, Schwabach, Germany). A buffer-salt mixture comprising 4 g anhydrous MgSO_4_, 1 g NaCl, 1 g C_6_H_5_Na_3_O_7_·2H_2_O, and 0.5 g disodium C_6_H_6_Na_2_O_7_·1.5H_2_O was then added, followed by immediate vigorous shaking for 1 min. To adjust the pH to 5.0–5.5, 5 mol dm^−3^ NaOH was added to acid-rich samples: 100 µL for strawberries, 400 µL for raspberries, and 800 µL for blackcurrants. The extract was centrifuged using a Hermle centrifuge (Hermle AG, Gosheim, Germany) at 6000 rpm for 5 min.

Post centrifugation, 6 mL of the acetonitrile phase was transferred to dispersive solid-phase extraction (DSPE) tubes containing 150 mg MgSO_4_, 25 mg primary secondary amine (PSA), and 2.5 mg graphitized carbon black (GCB). The tubes were shaken for 30 s (for PSA-only tubes) or 2 min (for tubes containing PSA and GCB) and then centrifuged at 6000 rpm for 5 min. A total of 990 µL of the purified extract and 10 µL of 5% formic acid were transferred to a vial and analyzed via LC-MS/MS.

### 2.3. LC-MS/MS Analysis

Internal standard triphenyl phosphate (TPP) (Merck Group, Darmstadt, Germany) was used for calibration, and pesticide standards were sourced from Dr. Ehrenstorfer GmbH (Augsburg, Germany). The standard stock solutions were prepared in acetonitrile or methanol, depending on the solubility of each individual compound, and stored at 4 °C in the dark. The working multi-compound standard solutions for matrix-matched calibrations and validation studies were prepared by appropriate dilution of the stock solutions [24,25].

The stock solutions were prepared for a concentration of 0.250 ng/µL (0.250 ppm) were prepared as follows: from the 1 mg/L standard solution, 0.250 mL was transferred into a brown vial and 0.750 mL of acetonitrile was added. The vial was labelled and stored in the dark in a freezer, with a validity of 12 months. Concentrations of 0.001, 0.005, 0.010, 0.020, 0.040, and 0.080 mg/L were prepared in a 2 mL vial by adding 4 µL of the 0.250 mg/L working standard solution and 50 µL of the 1 mg/L TFP working solution and completing the volume to 1 mL with 936, 920, 900, 860, and 780 µL of acetonitrile and 10 µL of 5% formic acid in acetonitrile.

The Shimadzu 8060 NEXERA LC system (Shimadzu Europa GmbH, Duisburg, Germany), equipped with two binary pumps, an autosampler, a column oven, and a detector, was coupled to a triple quadrupole MS system featuring an ion source. Chromatographic separation was performed on a Shimadzu Velox Biphenyl analytical column (2.1 × 100 mm, 1.8 µm particle size) at 40 °C. The mobile phase consisted of 10 mM ammonium acetate and 0.01% formic acid in ultrapure water (A) and 10 mM ammonium acetate and 0.01% formic acid in methanol (B). The gradient program began at 5% B, increased linearly to 90% B over 20 min, and was maintained at 98% B for 4 min. After a 24 min run time, a 6 min post-run equilibration at 5% B was applied. The flow rate was 0.4 mL min^−1^ and the injection volume was 5 µL. The electrospray ionization (ESI) source was operated in positive ion mode. Operating parameters included a gas temperature of 325 °C, a gas flow rate of 10 L min^−1^, and a nebulizer gas flow rate of 3 L min^−1^. Ion acquisition was performed in multiple-reaction-monitoring (MRM) mode. LabSolutions software Version 5.120 (Shimadzu Europa GmbH, Duisburg, Germany) was used for data acquisition, instrument control, and data analysis. Pesticide quantification was conducted via the internal standard method, with matrix-matched calibration applied to ensure accuracy.

Validation parameters are presented in Table 1, in accordance with DG/SANTE guidelines [26], and the combined uncertainty was calculated at the 0.01 mg/kg concentration level. All 74 pesticide compounds listed in Table 1 and Supplementary Materials S1 were included in the LC-MS/MS analytical method and were monitored across all 620 horticultural samples.

### 2.4. Data Processing and Analysis

Data processing utilized Python 3.13.2 (Python Software Foundation, Wilmington, DE, USA) [28] and pandas 2.2.3 (Seattle, WA, USA, WatchGuard Technologies) [29] to extract and clean structured datasets (947 rows × 78 columns) from an Excel file. Missing values were addressed via median/mean imputation or systematic removal of non-essential rows/columns. Exploratory Data Analysis (EDA) employed matplotlib 3.10.0 [29], seaborn 0.13.2 [30], and plotly/dash (“Plotly,” n.d.) for visualization, including hierarchical sunburst charts. Descriptive statistics (mean, median, min, max, standard deviation) were computed [31]. ANOVA (*p* < 0.05, statsmodels library) assessed pesticide usage differences across categorical variables (continent, product type, pesticide) while K-means clustering grouped datasets by similarity to identify usage patterns.

## 3. Results and Discussion

### 3.1. Pesticides Identification and Quantification by LC-MS/MS

The active ingredients identified in this study as residues in fruits and vegetables available on the Romanian market are classified by the WHO [27] as moderately hazardous (Class II), such as acetamiprid, clothianidin, difenoconazole, dimethoate, fenpyroximate, imazalil, imidacloprid, metalaxyl, pirimicarb, thiacloprid, and tebufenpyrad; slightly hazardous (Class III), such as bupirimate, buprofezin, clofentezin, dimethomorph, lufenuron, and pyrimethanil; and unlikely to present acute hazards in normal use, like azoxystrobin, boscalid, carbendazim, hexythiazox, pyriproxyfen, trifloxystrobin, and thiophanate-methyl.

The LC-MS/MS analysis of fruits and vegetables collected from Romanian retail markets highlighted multiple pesticide residues (Supplementary Materials S1). The Romanian samples were generally compliant with EU legislation [10], with identified residues including acetamiprid, boscalid, difenoconazole, and bupirimate. Multi-residue occurrences were common, mainly in leafy vegetables and soft fruits, where two to four pesticide residues were detected in several samples. These findings were consistent with the EFSA 2021 report, which stated that about 27% of European fruit and vegetable samples contained two or more pesticide residues [32].

Bupirimate, difenoconazole, and pyrimethanil were selected from the identified residues for detailed interpretation due to their relatively high frequency of occurrence and concentrations that, in some cases, came near the EU maximum residue limits (MRLs). These compounds are commonly used in both pre- and post-harvest treatments for fruits and vegetables, mainly in imported products, and possess toxicological profiles that warrant further attention. For example, bupirimate, a systemic fungicide mainly applied in powdery mildew controlling, was evidenced in strawberries (0.427 ± 0.214 mg kg^−1^), red Kapia peppers (0.225 ± 0.113 mg kg^−1^), nectarines (0.039 ± 0.019 mg kg^−1^), and golden apples (0.012 ± 0.006 mg kg^−1^). Their concentrations were below the EFSA MRLs (1.5 mg kg^−1^ for strawberries and sweet peppers and 0.3 mg kg^−1^ for apples and nectarines) [33]. Nevertheless, the residues detected in nectarines came close to the MRL threshold, indicating the necessity to optimize pre-harvest intervals and strict adherence to application guidelines [34].

Difenoconazole was identified in cherry tomatoes (0.693 ± 0.346 mg kg^−1^) and broccoli (0.349 ± 0.174 mg kg^−1^), their concentrations being below the respective MRLs (6 mg kg^−1^ and 1 mg kg^−1^) [33]. Nevertheless, the toxicological relevance should not be under-evaluated. The U.S. Environmental Protection Agency has labelled difenoconazole as a potential human carcinogen based on liver tumor formation in mice under chronic exposure [35].

Pyrimethanil, a fungicide with preventative and curative characteristics, was mainly detected in peppers, cucumbers, citrus fruits, and grapes. Concentrations varied widely, from 0.012 ± 0.006 mg kg^−1^ to 4.137 ± 2.068 mg kg^−1^, suggesting diverse treatment practices. High residue levels were mostly evidenced in imported samples, consistent with the literature, indicating more intensive chemical usage in non-EU agriculture [36]. While the EFSA does not categorize pyrimethanil as a carcinogen, cumulative exposure from sources has refined dietary intake evaluation for vulnerable consumers [8].

Compared to Romanian products, imported ones, mostly those from non-EU countries, presented higher residue diversity and concentration. Compounds such as azoxystrobin, boscalid, and acetamiprid were frequently detected in concentrations above 0.2 mg kg^−1^. On the other hand, North American imports commonly showed lower residue burdens (below 0.2 mg kg^−1^), reflecting more stringent pre-harvest intervals and more conservative pesticide regimes [37]. The results were in accordance with international surveillance data indicating variability in pesticide enforcement and agronomic protocols across trading regions [38].

Even though no sample exceeded the legal MRLs, some residues approached critical thresholds, or they are known to have toxicological concerns. The co-occurrence of pesticides with similar action mechanisms, such as neonicotinoids or triazoles, highlights the requirement for cumulative risk assessment protocols, as promoted by the EFSA’s PRIMo model [39].

### 3.2. Multi-Level Hierarchical Representation and Analysis of Variance (ANOVA) for the LC-MS/MS Data

**Figure 1:**
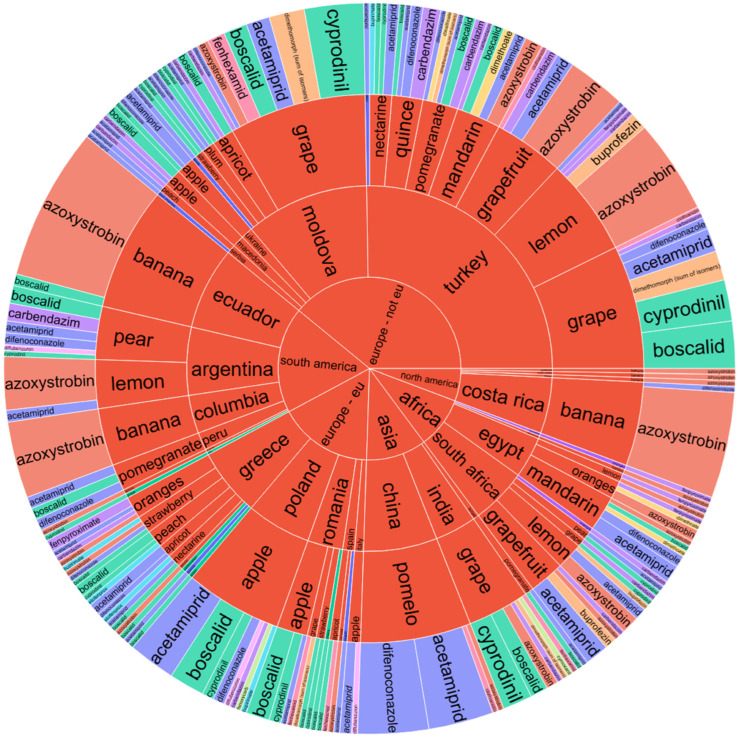
Sunburst chart for pesticide contamination of fruits depending on the continent and country of origin, product name, and pesticide type.

**Figure 2:**
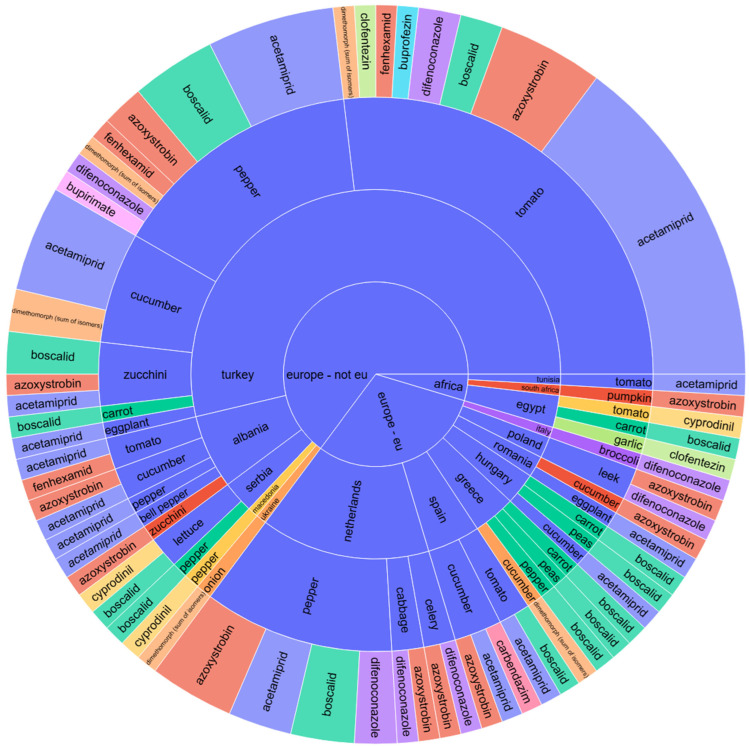
Sunburst chart for pesticide contamination of vegetables depending on the continent and country of origin, product name, and pesticide type.

Sunburst charts, as depicted for fruits (Figure 1) and vegetables (Figure 2), serve as multifaceted analytical tools facilitating the visualization of pesticide contamination across various dimensions including the continent, country of origin, product name, and pesticide type. Particularly, within Europe, the pesticide boscalid was prevalent in apples sourced from Romania and Poland. Meanwhile, strawberries and lemons from Greece have been documented to contain residues of bupirimate and azoxystrobin, indicating regional variances in pesticide application practices. Moreover, grapes imported from India were found to contain cyprodinil and boscalid, and pomelos from China exhibited residues of pesticides, including difenoconazole and acetamiprid.

The assessment of pesticide residue presence in fruits from both North and South America revealed that bananas originating from Costa Rica, Ecuador, and Colombia possessed significant amounts of azoxystrobin, which was also detected in greater concentrations in lemons from Argentina, as well as in oranges, lemons, and grapefruits from Africa. In examining the sunburst plot for fruit analysis, particular attention is drawn to non-European Union (EU) countries such as Moldova and Turkey, which exhibited elevated levels of contamination in grapes, lemons, and grapefruits with pesticides including boscalid, acetamiprid, cyprodinil, and azoxystrobin (Figure 1). However, statistical testing through ANOVA (described below) did not reveal significant differences in pesticide residue levels across continents for fruits (*p* = 1.000), nor for the pesticide type alone (*p* = 0.789), suggesting that the observed difference, although visually evident, was not statistically supported at the 0.05 significance level. Similarly, the interaction term (Continent × Pesticide) showed no significant effect (*p* = 0.104).

Notably, these pesticide residues are similarly present in the vegetable category, affecting peppers, tomatoes, cucumbers, zucchinis, and lettuce from non-EU nations such as Turkey, Albania, and Serbia.

A comparative analysis of the sunburst plot for vegetables (Figure 2) reveals a persistent presence of these pesticide residues across the various studied vegetable types, sourced from both EU countries and Asia. For vegetables, the pesticide type showed a near-significant effect (*p* = 0.061), suggesting some variability in residue levels depending on the pesticide used, while no statistical significance was observed for continent or the interaction term (*p* = 0.911 and 0.712, respectively). Therefore, although descriptive analysis pointed toward regional residue trends (e.g., tomatoes from Turkey with difenoconazole and acetamiprid), these did not translate into statistically significant differences.

A detailed examination of Figure 1 revealed different pesticide contamination patterns in fruits depending on their geographical origins. Within the European Union, Romania and Poland were prominent for their apple exports, which often contained boscalid. Greece showed significant concentrations of bupirimate in strawberries and azoxystrobin within citrus fruits, such as lemons and mandarins, evidencing common Mediterranean crop protection practices targeting mildew and storage diseases.

Non-EU European countries, including Moldova and Turkey, showed elevated residue levels in grapes, lemons, and grapefruits. Boscalid, acetamiprid, cyprodinil, and azoxystrobin were frequently detected, suggesting intensive pest control regimes and less harmonized regulatory enforcement compared to EU standards. China (Asia) showed notable detection of difenoconazole and acetamiprid in pomelos while India (Asia) contributed to high occurrences of boscalid and cyprodinil in grapes. These findings indicate post-harvest and systemic treatments targeting export quality and shelf life.

South American countries (Argentina, Ecuador, and Colombia) often export bananas, lemons, and grapefruits with azoxystrobin residues. African imports, especially from Egypt and South Africa, revealed pyrimethanil, imazalil, and azoxystrobin residues in citrus fruits, pointing to widespread post-harvest fungicide applications. In some cases, concentrations targeted MRL thresholds, indicating the need for tight residue control.

A recurring pattern evidenced in Figure 1 was the prevalence of azoxystrobin across multiple fruit types and continents, mainly in bananas, lemons, and grapefruits. This reflected its global role in managing fungal pathogens, including black sigatoka and storage mold. Moreover, fruits originating from non-EU countries presented a higher residue diversity and frequency of multi-residue detection compared to EU-origin samples. This supports the idea that variation in agricultural protocols and enforcement mechanisms contributes to more complex residue profiles in imports.

The hierarchical visualization in Figure 2 indicates the trend in pesticide residues across various vegetables. EU countries, such as Spain and Romania, were linked with tomatoes and peppers containing acetamiprid, boscalid, and difenoconazole residues. Spanish cucumbers had the highest single-compound finding of carbendazim, consistent with intensive pesticide use in greenhouse cultivation. Romanian vegetables, especially peppers and tomatoes, also presented multi-residue profiles, reflecting domestic protection protocols against fungal and insect threats.

On the contrary, Turkey and Albania (non-EU Balkan countries) were linked with higher pesticide residues in tomatoes, cucumbers, and lettuce. Clofentezin, acetamiprid, difenoconazole, and azoxystrobin were repeatedly detected in Turkish vegetables, evidencing a multi-modal chemical control regime commonly used in greenhouse production. Albanian cucumbers contained pyrimethanil and boscalid, consistent with regional pest control practices in cucurbit crops.

Egyptian tomatoes displayed residues of cyprodinil, indicative of post-harvest mold control protocols typical of North African export chains. Vegetables from Asia demonstrated low-level residues, such as dimethoate, thiacloprid, and pyrimethanil, indicating fragmented production and variable pesticide regimes.

Overall, Figure 2 highlights that vegetables, especially tomatoes, peppers, and cucumbers, frequently presented multi-residue profiles more frequently than fruits.

While the identified pesticides were consistent in higher concentrations across different continents, discernible variations existed in the phytosanitary treatments applied to distinct vegetable species, which could vary significantly from one country to another. For instance, tomatoes from Turkey were characterized by the presence of difenoconazole, acetamiprid, azoxystrobin, and boscalid, along with lower concentrations of clofentezin residues. In contrast, tomatoes from Spain displayed only residues of acetamiprid and boscalid, whereas those imported from Egypt revealed solely cyprodinil residues.

The systematic LC-MS/MS screening of vegetables purchased from Romania market revealed persistent yet low-level pesticide residues (below 0.10 mg kg^−1^ for more than 80% of the samples) but clear geographical signatures. It was observed that sweet peppers contained carbendazim at 0.078 ± 0.014 mg kg^−1^ and chlorothalonil at 0.062 ± 0.009 mg kg^−1^, confirming earlier observations that Turkish greenhouse crops rely on benzimidazole and chloronitrile fungicides for grey-mold control [40]. Tomatoes evidenced a multi-residue profile of acetamiprid (0.037 ± 0.008 mg kg^−1^), azoxystrobin (0.031 ± 0.006 mg kg^−1^), clofentezin (0.028 ± 0.005 mg kg^−1^), and difenoconazole (0.043 ± 0.011 mg kg^−1^), highlighting alternated fungicide/insecticide programs used for tomato leaf-mold and Tuta absoluta management [41].

Albanian cucumbers contained pyrimethanil at 0.055 ± 0.012 mg kg^−1^ and trace boscalid at 0.024 ± 0.004 mg kg^−1^, evidencing the strategy reported for Balkan cucurbits [42].

Spanish cucumbers exhibited the highest single-compound finding: carbendazim at 0.112 ± 0.027 mg kg^−1^, under the EU MRL (1 mg kg^−1^) but consistent with intensive winter greenhouse use [43]. Spanish tomatoes were clean enough, containing only acetamiprid (0.029 ± 0.007 mg kg^−1^) and boscalid (0.026 ± 0.006 mg kg^−1^), both below their MRLs (0.5–1 mg kg^−1^), being in accordance with Spain’s recent movement toward biocontrol in solanaceous crops [44].

Regarding non-EU vegetables, Egyptian tomatoes contained cyprodinil (0.047 ± 0.010 mg kg^−1^), a residue resulting from post-harvest grey-mold suppression in North-African supply chains [45].

Generally, the dataset emphasized heterogeneous residue fingerprints driven by regional pest spectra and regulatory environments. It was observed that the concentrations conformed with EU Regulation (EC) 396/2005; however, the co-occurrence of triazoles and neonicotinoids across several products reinforces the necessity for cumulative-risk strategies, mainly for consumers with increased tomato- or cucumber-based dietary regimes [40].

For other fruits and vegetables, as well as their respective countries of origin, pesticide concentrations detected via LC-MS were below 0.1 mg kg^−1^, indicating lower levels of contamination.

This analytical approach not only provides a visually compelling method for examining the geographical and categorical distribution of pesticide application but also enhances the ability to identify patterns or anomalies and thereby enhance our understanding of the geographical and categorical distribution of pesticide application.

The widespread use of pesticide residues in both urban and agricultural settings has raised concerns about their potential negative impacts on human and wildlife health. The use of the key terms “pesticide toxicity” in the Web of Science database revealed a substantial body of scientific literature investigating the toxicity of pesticides, including their effects on health and the environment, risk assessment tools, and progress in reducing pesticide use (25,597 scientific works as of 5 January 2025). Notably, 3082 papers have specifically addressed the effects of long-term pesticide exposure (keywords used: “Pesticides long-term exposure”).

**Figure 3:**
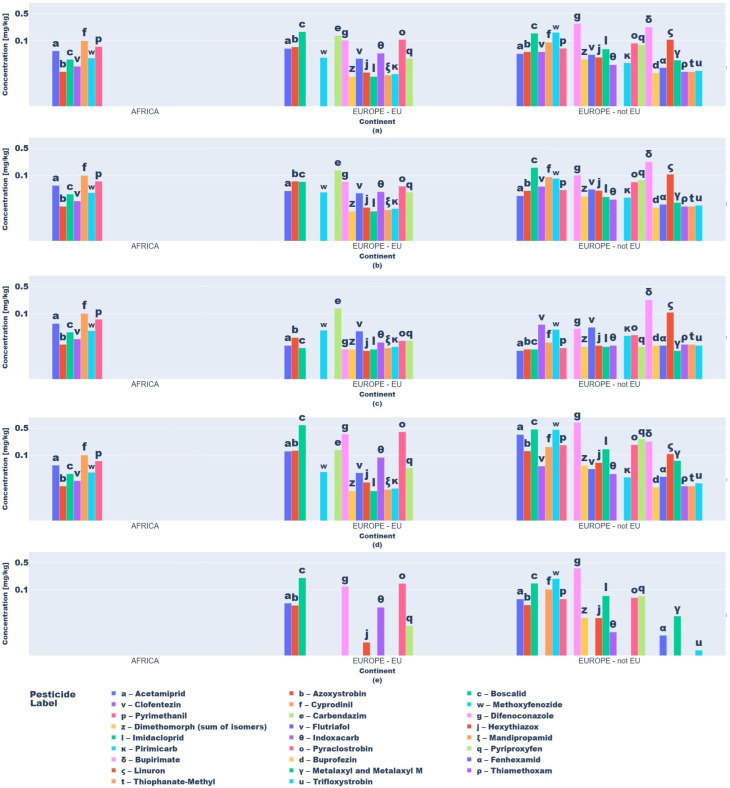
Key descriptive statistics of pesticide values in vegetable product type samples: (**a**) mean values; (**b**) median values; (**c**) minimum values; (**d**) maximum values; (**e**) standard deviation values.

**Figure 4:**
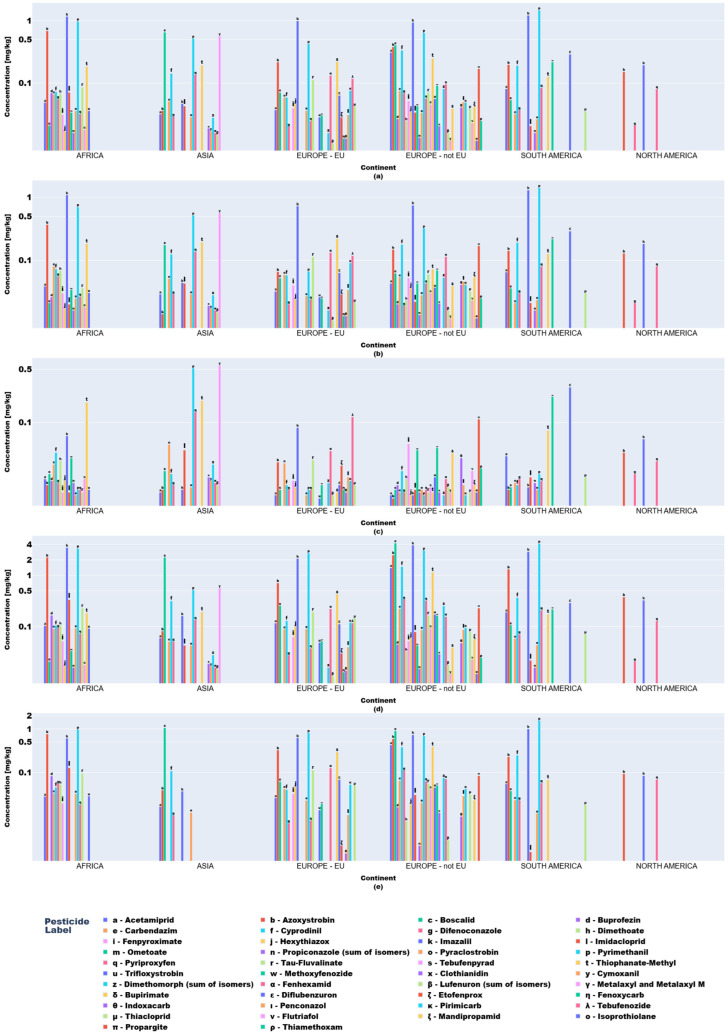
Key descriptive statistics of pesticide values in fruit product type samples: (**a**) mean values; (**b**) median values; (**c**) minimum values; (**d**) maximum values; (**e**) standard deviation values.

Descriptive statistics and visual analytics (bar charts, box plots) yielded data to map global pesticide usage patterns across continents, product types, and specific pesticides. Fruit and vegetable pesticide metrics (mean, median, min/max, SD), revealing central tendencies and extremes, require regulatory attention. High variability in application practices underscores disparities, with outliers indicating farms exceeding norms, raising concerns over resistance and health risks (Figure 3 and Figure 4).

Box plots (Figure 3 and Figure 4) delineated statistical trends. African vegetables exhibited uniform pesticide use (null standard deviation), dominated by cyprodinil, pyrimethanil, and azoxystrobin. EU vegetables showed variability, with higher mean residues (0.1–0.2 mg kg^−1^) for difenoconazole, pyraclostrobin, and boscalid and medians (0.05–0.15 mg kg^−1^) indicating intensive usage.

Non-EU vegetables exhibited elevated mean residues (>0.2 mg kg^−1^ for difenoconazole, bupirimate; 0.1–0.2 mg kg^−1^ for methoxyphenozide, boscalid, hexythiazox), with difenoconazole showing the highest variability (high standard deviation) due to divergent agricultural practices and regulatory frameworks.

Regionally, azoxystrobin, imazalil, and pyrimethanil dominated African fruits while Asian samples showed boscalid, imazalil, and thiacloprid. In Europe (EU/non-EU) and South America, imazalil and pyrimethanil were prevalent. Imazalil residues in citrus fruits (e.g., grapefruits: 0.058–3.855 mg kg^−1^; lemons: 0.062–3.451 mg kg^−1^) exceeded EFSA MRLs [12] for bananas (0.01 mg kg^−1^) and approached limits for grapefruits (4 mg kg^−1^).

These findings underscore significant regional disparities in pesticide use, necessitating tailored agricultural and regulatory strategies to address public health and sustainability challenges in Africa, Asia, and Europe.

The statistical analysis of pesticide usage was conducted through an ANOVA (analysis of variance) model built using the stats model’s library in Python, where the notation C(…) was employed to explicitly designate variables as categorical (Table 2). This modelling approach ensured that variables such as continent, product type, and pesticide name were treated as discrete groupings rather than continuous quantities, thereby allowing the model to assess how these distinct categories influenced the dependent variable—pesticide usage—measured as a continuous numerical outcome. In this framework, the ANOVA model examined the degree to which variation in pesticide usage can be attributed to each of these categorical factors and their interactions.

The resulting ANOVA table contains several key components necessary for interpreting the influence of each factor. The column labelled “sum sq” refers to the sum of squares, which quantifies the total variability in the data that is attributable to each factor or source, effectively capturing how much the observed values deviate from the overall mean. The “df” column denotes the degrees of freedom, representing the number of independent comparisons associated with each term in the model, and it reflects the complexity of the factor being tested. The F column reports the F-statistic, calculated as the ratio of the mean square of a given factor to the mean square of the residuals, which serves as a test statistic to evaluate whether the variation associated with that factor is significantly greater than expected under the assumption of random variation. Finally, the PR(>F) column presents the *p*-value corresponding to each F-test, which indicates the probability that the observed F-statistic could have occurred under the null hypothesis of no effect; lower *p*-values suggest stronger evidence against the null hypothesis and support the conclusion that the factor in question has had a statistically significant impact.

It was observed that all *p*-values were higher than 0.05, indicating that no statistically significant differences were detected for the examined factors. However, the use of ANOVA in this context remained fully justified. The analysis of variance was performed to assess whether the continent of origin, pesticide type, or their interaction had had a statistically significant effect on the pesticide residue levels observed in fruits and vegetables. Although no significant effects were found, this outcome was meaningful in itself and provides valuable insight: it suggests a lack of systematic variation in pesticide residues by continent or compound within the studied samples. It must be re-iterated that all 620 samples were collected from Romanian supermarkets, where sourcing is driven by centralized procurement contracts and commercial supply chains. These systems prioritize consistency and may therefore homogenize pesticide residue levels across different countries and continents. Moreover, the dataset reflected seasonal product availability, meaning that certain produce types had been imported from limited geographic sources, reducing within-group variability and statistical power. Some pesticide–continent combinations contained only a few samples, limiting the ability of the ANOVA model to detect subtle differences. Despite the lack of statistical significance, the application of ANOVA remains valid for hypothesis testing and methodological transparency. It demonstrates that, under current market requirements, residue levels in supermarket products were relatively uniform, with little variation attributed to the assessed categorical variables.

The analysis indicated that the variable C(Continent) did not exert a significant influence on pesticide residues, as evidenced by a *p*-value (PR(>F)) of 0.061 for the pesticide index, which approached the conventional threshold of significance (0.05). This suggests a potential, albeit inconclusive, effect of the type of pesticide on residue levels. Furthermore, the interaction between continent and pesticide (C(Continent):C(Pesticide)) produced a PR(>F) value of 0.712, indicating a lack of significant impact on residue levels.

The ANOVA results reported PR(>F) values of 1.000 and 0.789 for C(Continent) and C(Pesticide), respectively, suggesting that there was no significant correlation between the continent and pesticide type with respect to their effects on residue levels in fruits. Additionally, a PR(>F) value of 0.104 reflected an insignificant interaction between continent and pesticide.

The sum-of-squares (sum sq) values of 1.373 for vegetables and 301.324 for fruits indicated a noteworthy degree of unexplained variability attributable to the examined variables. This suggests that other factors, apart from continent and pesticide type, may significantly contribute to the presence of pesticide residues in fruits and vegetables. These findings highlight the need for further investigation into additional variables that may influence pesticide residue levels, thereby enhancing our understanding of agricultural practices and environmental safety.

**Figure 5:**
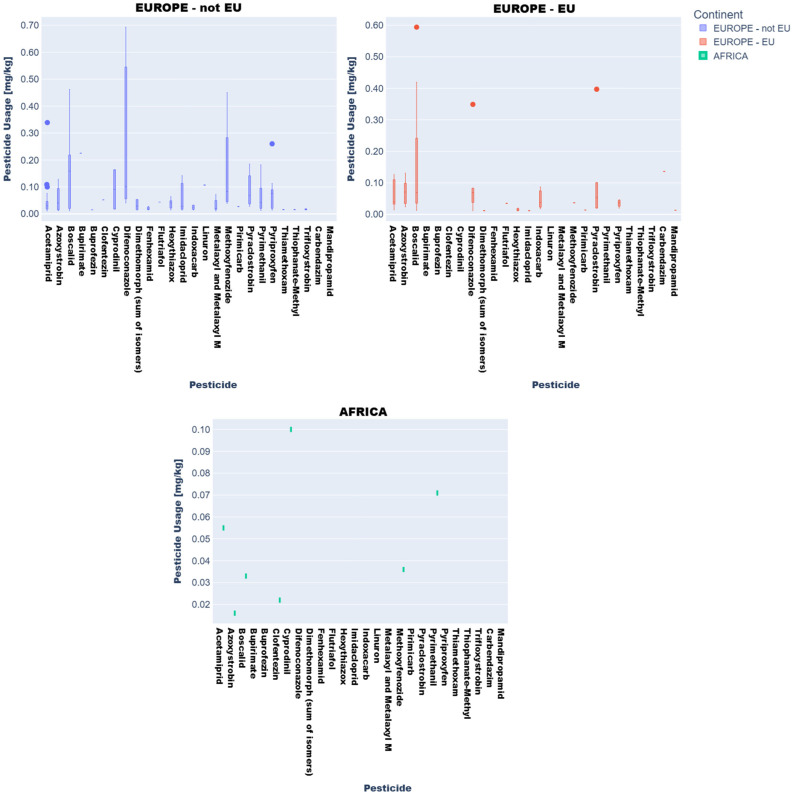
ANOVA results; box plots and bar charts for vegetables.

**Figure 6:**
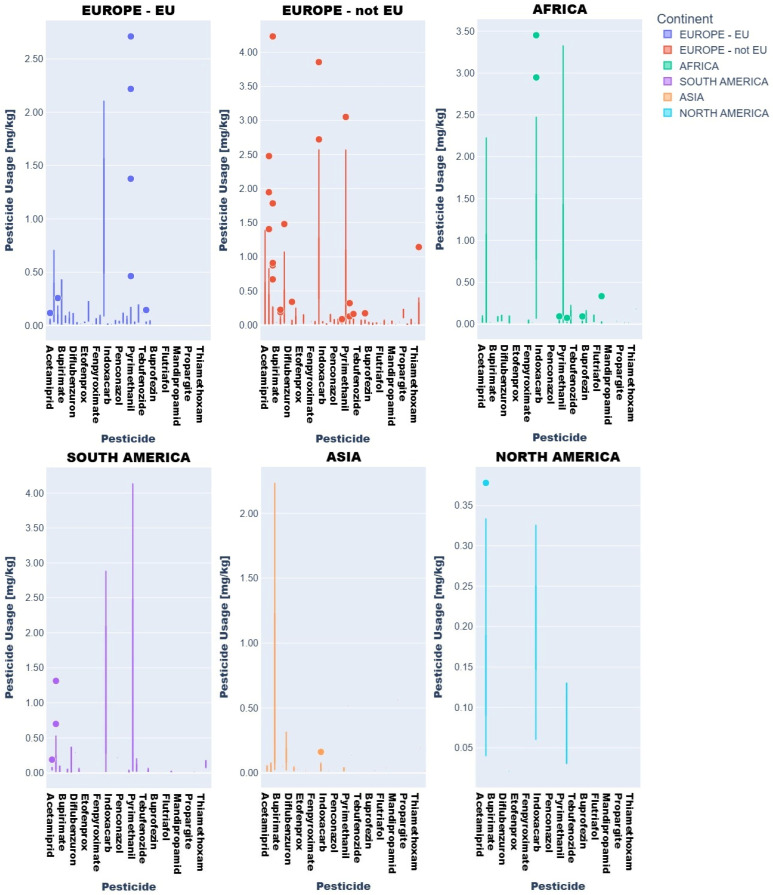
ANOVA results; box plots and bar charts for fruits.

The ANOVA results presented through a combination of box plots and bar charts were particularly effective in illustrating the distribution of pesticide usage across various groups, such as continents and product types, whereas bar charts succinctly summarize key statistical metrics including the F-statistic and *p*-value (Figure 5 and Figure 6).

The ANOVA analysis highlighted significant variability in pesticide residue concentrations throughout the fruit and vegetable samples (Table 3).

For example, in fruits, acetamiprid and azoxystrobin displayed the highest frequencies of outliers, with 12 and 13 instances, respectively (Table 3). These findings indicated that some fruit samples had abnormally high levels of these pesticides, a potential sign of excessive or inconsistent usage during cultivation or post-harvest treatment. Boscalid also manifested high variability, with six outliers detected in fruits. In vegetables, although the number of outliers was generally lower, acetamiprid (four outliers) and boscalid (three outliers) remained among the most often observed, pointing to isolated cases of increased residue levels. Other pesticides (imidacloprid, pyraclostrobin, and pyrimethanil) revealed one outlier each in the vegetables, indicating the sporadic occurrence of high concentrations.

These findings highlighted the variability in pesticide residue concentrations among various commodity types, reinforcing the necessity for ongoing monitoring and regulatory governance. The detection of multiple outliers, mainly in fruits, revealed a potential request for more rigorous compliance with good agricultural practices (GAPs) and a reassessment of current pesticide application protocols. The integrated application of ANOVA and outlier analysis demonstrated an efficient strategy to identify samples that may have presented increased food safety concerns due to atypical residue profiles.

### 3.3. Integration of Python Analytics with LC-MS/MS Data and Elucidation of Contamination Patterns

**Figure 7:**
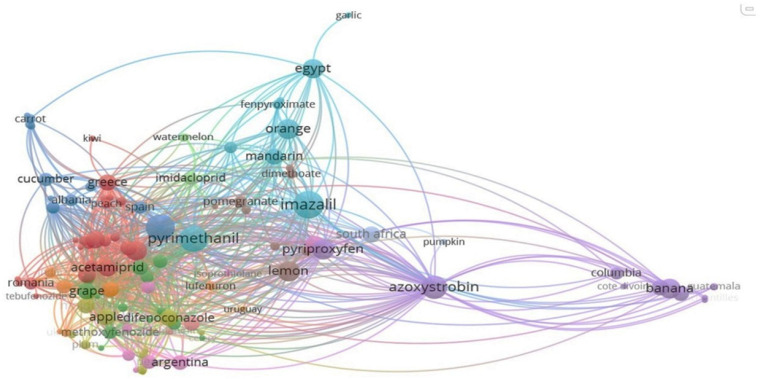
K-means clustering of pesticide residue in fruits and vegetables available in Romanian markets.

Considering the substantial dataset at hand, the application of K-means clustering was employed to group the datasets into clusters based on similarity, with each data point assigned to the cluster that was nearest to its mean. Unlike ANOVA, which tests for statistical differences among groups, K-means clustering uncovers patterns or groupings within the data based on inherent similarities (Figure 7). This methodological choice facilitated a deeper understanding of the underlying relationships in pesticide usage patterns across various farms.

The analysis of pesticide occurrence across 12 clusters (Figure 7) revealed systematic pesticide–crop–trade linkages, driven by phytosanitary needs, climatic factors, and EU regulatory compliance. Clusters 1–3 highlighted fungicides (pyraclostrobin, boscalid) and insecticides (acetamiprid) dominating European fruit/vegetable imports (e.g., strawberries, grapes), with residues reflecting intensive use in Greece, Romania, and Serbia. Cluster 2 emphasized hexythiazox (toxic to aquatic invertebrates) and methoxyfenozide (persistent in soil) [46], both of low risk to humans but ecologically concerning.

Clusters 4–6 demonstrated regional specialization: difenoconazole in Eastern European apples/plums (Cluster 4), tropical fungicides (azoxystrobin) and insect growth regulators (pyriproxyfen) in banana exports (Cluster 5), and post-harvest fungicides (imazalil, pyrimethanil) in Egyptian citrus (Cluster 6). Clusters 7–12 reflected targeted applications: dimethomorph in Indian/Ukrainian vegetables (Cluster 7), insecticides (buprofezin, dimethoate) in Israeli/Peruvian citrus (Cluster 8), and neonicotinoids (clothianidin, imidacloprid) in Chinese pomelo and Brazilian watermelon (Clusters 10–11). Key distinctions included fungicide dominance in temperate crops (e.g., Clusters 1, 4, 9) versus insecticide reliance in tropical/horticultural systems (e.g., Clusters 5, 8). Export-driven patterns (Turkey’s vegetables, Moldova’s apples) underscored trade’s role in residue profiles. Cluster 12 weakly linked South Africa to pumpkin trade, lacking strong pesticide ties.

Within Cluster 1 (Supplementary Materials S2—Table S1 and Figure S1) was evidenced the dominance of pyraclostrobin (73 occurrences), boscalid (52 occurrences), and acetamiprid (47 occurrences), pesticides that were intensively used in crop protection, mainly for combating fungal diseases and specific pests. This explained the frequent detection of these residues in imported fruit samples, given both their high chemical persistence and their systematic application according to the phytosanitary needs of each crop and the European regulations on maximum residue limits.

The frequent co-occurrence of these pesticides in strawberries, peaches, and cherries, mainly from Greece (thirty-one cases), Romania (ten), and Serbia (five), evidenced regional crop-protection patterns and trade-driven exposure. This reflected how local agronomic needs, pest pressure, and intensive treatments converged with the EU food-safety rules. The distribution of the identified pesticides indicated a prevalence of fungicides, such as pyraclostrobin and boscalid, commonly used to prevent and control fungal infections in fruits and vegetables [47], along with acetamiprid, a systemic insecticide repeatedly detected in the analyzed samples, reflecting both the different crop protection strategies adopted according to the type of agricultural product and its vulnerability to diseases and pests. Also, it reflected the variations in pesticide application regimes influenced by climatic conditions, local standards, and the requirements imposed by European markets, underscoring the importance of continuous monitoring to ensure that imported products comply with the European Union’s food safety regulations.

For instance, the commercial formulation of acetamiprid, a neonicotinoid insecticide, exhibits genotoxic effects on human peripheral blood lymphocytes, evidenced by increased sister chromatid exchanges, chromosomal aberrations, and micronucleus formation [48]. Acetamiprid has significantly decreased cell viability; induced reactive oxygen species (ROS) generation, lipid peroxidation, and DNA damage; triggered caspase-dependent apoptosis in PC12 cells, a rat pheochromocytoma adrenal medulla cell line [49]; and increased biomarkers of genotoxicity in the myocardium in male rats through oxidative stress-mediated DNA damage and the dysregulation of DNA repair and cell cycle pathways [50]. Another study investigated the species-specific neurotoxic effects of sub-lethal doses of acetamiprid on the neuroblasts of *Drosophila melanogaster* and *Drosophila suzukii*. The results showed that acetamiprid, similar to nicotine, caused genotoxic damage and decreased mobility in both species, with *D. suzukii* being more sensitive and susceptible to even lower doses [51].

Boscalid, one the most widely used broad-spectrum fungicides, a succinate dehydrogenase inhibitor (SDHI), has been frequently found in various environmental samples, including sediments, water, and aquatic organisms, due to its slow degradation, raising concerns about its presence in ecosystems and potential exceeding of drinking water pesticide thresholds. In this study, higher concentrations of boscalid residues (4.231 ± 2.115 mg kg^−1^) were found in rose grapes and red Kapia pepper (0.594 ± 0.297 mg kg^−1^). In apples, boscalid residue levels ranged from 0.019 ± 0.010 mg kg^−1^ to 0.115 ± 0.058 mg kg^−1^. Notably, these residues approached the maximum residue levels (MRLs) established by Commission Regulation (EU) 2022/1324 [52] for grapes (5 mg kg^−1^) but remained well below the MRLs stipulated for sweet peppers (3 mg kg^−1^) and apples (2 mg kg^−1^).

Expanding on the potential impacts of boscalid, several studies investigated the effects of short-term exposure to this succinate dehydrogenase inhibitor (SDHI) and its mixture with other pesticides. Evaluating the effects of Boscalid and Bixafen across peripheral blood mononuclear cells (PBMCs), HepG2 liver cells, and BJ-fibroblasts, we found a significant decrease in the oxygen consumption rate and an increase in the number of early apoptotic cells and in mitochondrial superoxide levels in HepG2, suggesting a direct implication of mitochondrial dysfunction induced by these compounds in human cells. In another study, a mixture of the same concentrations of pyraclostrobin and boscalid impacted mitochondrial function in human hepatocytes, leading to decreased oxygen consumption, increased mitochondrial superoxide levels, reduced ATP content, and lower cell viability, raising concerns about the selectivity and safety in agricultural use of these pesticides [53]. Furthermore, exposure to environmentally relevant concentrations of the persistent fungicide boscalid has significantly impaired the neurobehavioral responses of zebrafish, including alterations in locomotion, phototaxis, and predation, and affected gene expression related to neurodevelopment and visual function, suggesting potential neurotoxic mechanisms [54]. Recent research had found that prolonged exposure to the fungicide boscalid significantly increased honeybee mortality and revealed a unique pattern of reinforced toxicity after 17–18 days, emphasizing the need for time-to-death experiments to assess chronic toxicity [55]. Another study demonstrated that the fungicide Pristine^®^, which contains 25.2% boscalid and 12.8% pyraclostrobin, negatively impacts honey bee pollen consumption and survival, raising concerns that standard laboratory tests may inadequately predict the harmful effects of pesticides on pollinators in real-world scenarios [56].

Cluster 2 (Supplementary Materials S2—Table S1 and Figure S2) marked an extensive usage of hexythiazox (17 occurrences) and methoxyfenozide (15 occurrences) for fruit and vegetable protection, particularly against mites and Lepidoptera pests, reinforcing the role of chemical control strategies in maintaining crop health and productivity.

A major distinction between the two clusters was the type of predominant pesticides: Cluster 1 was marked by a high occurrence of fungicides like pyraclostrobin and boscalid, commonly used for managing diseases in strawberries, peaches, and cherries. On the contrary, Cluster 2 was distinguished by the dominance of hexythiazox and methoxyfenozide, primarily employed to control mites and insect pests in grapes and tomatoes. This differentiation underscored how regional climate conditions, pest pressure, and regulatory frameworks shape pesticide usage patterns across international trade networks, affecting the frequency of pesticide detections in imported food products.

In 2017, the U.S. Environmental Protection Agency reported that hexythiazox, an ovicide/miticide for spider mites on crops and ornamental plants, is highly toxic to freshwater aquatic invertebrates, having an acute EC_50_ of 0.74 ppm and a chronic NOAEC (No-Observed-Adverse-Effect Concentration) of 6.1 ppb for daphnids [46]. However, studies on mollusks, marine fish, avian reproduction, and aquatic plants were deemed unnecessary as existing data indicated low acute risks (e.g., bluegill LC_50_ = 0.53 ppm mitigated by low-use rates), no unique toxic mechanisms (e.g., calcium disruption in mollusks), and no adverse effects in vertebrate studies (rat/avian reproduction). Ten years later, the toxicology database was completed by the EPA, with primary target organs identified as the adrenals and liver in chronic animal studies, though hexythiazox showed no developmental, reproductive, neurotoxic, or mutagenic effects. Classified as “Likely to be Carcinogenic to Humans” due to liver tumors in mice and mammary tumors in rats, carcinogenic risk is addressed via a non-linear reference dose (RfD) approach deemed sufficient to account for chronic toxicity. Acute toxicity in humans is low (Category IV for oral/dermal/inhalation routes), with mild eye irritation (Category III). Dietary exposure assessments, using conservative assumptions (tolerance-level residues, 100% crop-treated), indicate chronic risks at 23% of the chronic population-adjusted dose for the general population and 97% for children 1–2 years old, below levels of concern. Occupational handler risks (inhalation) can be mitigated by mandatory personal protective equipment, yielding margins of exposure (MOEs) from 22,000 to 5.6 million, far exceeding the 100 thresholds. Residential post-application exposures (e.g., turf contact) show no risks, with MOEs >10,000. The EPA has concluded no significant dietary, aggregate, or occupational risks, supporting label amendments for proposed uses [57].

Methoxyfenozide, an insecticide used on crops such as grapes, maize, vegetables, and leafy greens, exhibits low acute toxicity (oral, dermal, inhalation) and is classified as “Likely Carcinogenic to Humans” (Category 2) due to thyroid and liver tumors in rats [35]. Chronic toxicity endpoints led, in this study, to an acceptable daily intake (ADI) and acute reference dose (ARfD) of 0.1 mg kg^−1^ body weight/day. The key concerns include unresolved endocrine disruption potential, incomplete metabolism studies, and insufficient data on impurities (e.g., RH-116267). Consumer dietary risk assessments for proposed maximum residue levels (MRLs) on grape leaves and sweet corn have shown no chronic risks, though acute exposure concerns have been noted for crops like lettuce and cabbage. Environmental assessments have highlighted methoxyfenozide’s persistence in soil (DT50 > 1000 days) and groundwater contamination risks for its metabolite RH-131154 (M08), exceeding the 0.1 μg L^−1^ threshold. Ecotoxicological data gaps include chronic risks to aquatic organisms and effects on honeybee larvae and non-target arthropods (particularly *Lepidoptera*). It has been concluded that while current uses pose no critical risks, unresolved issues—such as endocrine activity, groundwater contamination, and ecological impacts—require additional data [58].

Cluster 3 (Supplementary Materials S2—Table S1 and Figure S3) revealed a regional specialization in crop protection strategies aimed at addressing both fungal diseases and weed management. The co-occurrence of metalaxyl (eleven occurrences) and linuron (one occurrence) within some crops (such as cucumber, carrot, and clementine, predominantly associated with exports from Turkey and Spain) reflected a geographically structured agronomic model like Cluster 1 and Cluster 2, where pesticide use was shaped by climatic conditions, crop susceptibility, and trade dynamics. Cluster 3 was differentiated through the significant presence of herbicides and systemic fungicides, particularly metalaxyl, used to combat soil-borne diseases. One of the features of Cluster 3 was the frequency of Turkey (201 occurrences), indicating a major role in the export of vegetables, particularly cucumbers and zucchini, requiring intensive disease management to lead a high frequency of pesticide detections.

A key regional aspect of Cluster 4 (Supplementary Materials S2—Table S1 and Figure S4) was the strong representation of Moldova (32 occurrences) and Poland (20 occurrences), suggesting that these countries required extensive fungicide and insecticide applications. The high detection frequency of difenoconazole (40 occurrences), particularly in apple and plum production, highlighted a regulated yet intensive use of this fungicide in Eastern European agricultural systems, consistent with the residue patterns observed across multiple clusters.

Cluster 5 (Supplementary Materials S2—Table S1 and Figure S5) highlighted a distinct concentration of tropical agricultural products and fungicidal/insecticidal applications, mainly linked to banana production and major exporting countries such as Colombia, Costa Rica, Ecuador, and Mexico. The high frequency of azoxystrobin (137 occurrences)—intensively applied for banana plantations to control Black Sigatoka, along with pyriproxyfen (85 occurrences)—an insect growth regulator targeting pests like whiteflies and scale insects common in tropical climates, underscored a strong correlation between fungicides and insect growth regulators in tropical crop management. This pattern aligned with the specialized pesticide usage observed within Clusters 1–4, where fungicides and insecticides were concentrated on fruit and vegetable protection strategies. The presence of Côte d’Ivoire, Guatemala, and the French Antilles, although with lower occurrences, indicated a secondary role in banana production and export, reinforcing the structured trade dependencies that shape pesticide application strategies.

According to the United States Environmental Protection Agency, azoxystrobin is a widely used strobilurin pesticide applied to combat fungal pathogens, having low application rates, long intervals, a broad control spectrum, low toxicity to humans, and non-target and high-toxicity risks to freshwater and marine organisms [59]. In an extensive review, the concerning presence and effects of azoxystrobin in aquatic ecosystems were evidenced, mainly targeting the potential toxicity risks to non-target aquatic organisms based on its action mode [57]. Azoxystrobin targets the mitochondrial respiratory complex III (also known as cytochrome c oxidoreductase or the cytochrome bc1 complex) and inhibits electron transfer between cytochrome b and cytochrome c1 at the ubiquinol oxidizing site (Qo) of this complex, leading to an excess of electrons escaping from the mitochondrial respiratory chain, which triggers cellular oxidative stress and the abnormal generation of reactive oxygen species (ROS) [60]. The potential transfer from mothers to offspring was also assessed in a study, highlighting the neurotoxic effects of azoxystrobin acid as a metabolite of azoxystrobin [61]. Azoxystrobin acid was detectable in all urine samples from pregnant women and 70% of children’s samples, with higher concentrations noted in children [48].

The strong co-occurrence of imazalil (245 occurrences) and pyrimethanil (207 occurrences) in oranges and mandarins suggested that Cluster 6 (Supplementary Materials S2—Table S1 and Figure S6) primarily represented citrus production and post-harvest protection strategies, mainly in North Africa and Southern Africa, with Egypt (70 occurrences) playing a dominant role in citrus exports. Garlic’s presence in Cluster 6 was probably due to trade overlaps with citrus-exporting countries like Egypt rather than a direct pesticide usage similarity. The presence of tau-fluvalinate (14 occurrences), an insecticide targeting mites and pests in citrus orchards, along with fenpyroximate (6 occurrences), another acaricide used in integrated pest management programs, further reinforced the specialized pest control strategies tailored to citrus production.

Pyrimethanil is a potent phenylamino-pyrimidine fungicide widely used in agriculture to control gray mold and other fungal diseases by inhibiting key enzymes involved in methionine biosynthesis and cell wall degradation. Pyrimethanil inhibits the growth of primary producers like macroalgae and microalgae and affects the survival, reproduction, and growth of various aquatic vertebrate species, disrupting thyroid–pituitary homeostasis in rodents and affecting the liver and thyroid in mammals. Long-term exposure to pyrimethanil also disrupts habitats; decreases genetic diversity in certain insect populations; causes detrimental morphological changes in amphibians; promotes the formation of αβ peptides, microglial proliferation, and neuronal loss in mice; and may contribute to the onset or progression of neurodegenerative diseases [62].

The co-occurrence of dimethomorph (49 occurrences), lufenuron (33 occurrences), and cymoxanil in onions—Cluster 7 (Supplementary Materials S2—Table S2 and Figure S7) represented a specific focus on fungicides used in vegetable production, particularly for downy mildew control. A notable feature of Cluster 7 was the presence of India (seven occurrences) and Ukraine (three occurrences), suggesting that pesticide usage patterns in this cluster was linked to international trade in both raw agricultural products and agrochemical formulations. The inclusion of Uruguay (one occurrence), although weak, indicated secondary trade relationships and shared pesticide regulations.

The dominance of lemon (eighty-four occurrences) and pomegranate (eleven occurrences), associated with buprofezin (five occurrences) and dimethoate (five occurrences), in Cluster 8 (Supplementary Materials S2—Table S2 and Figure S8) suggested a focus on insect pest management in citrus and pomegranate cultivation. This cluster represented an agricultural model focused on insect control, mainly in high-value fruit crops. The presence of Israel (one occurrence) and Peru (four occurrences) suggested that pesticide application in this cluster was closely tied to trade and export activities involving these countries. Additionally, the inclusion of ometoate (two occurrences) and tebufenpyrad (four occurrences) reinforced the emphasis on insecticides and acaricides, commonly used in citrus and pomegranate orchards.

Cluster 9 (Supplementary Materials S2—Table S2 and Figure S9) highlighted a structured association between fungicidal and insecticidal applications in pome and citrus fruit production, including key exporting countries such as Argentina and Portugal. The dominance of grapefruit (47 occurrences) and pear (21 occurrences), linked with thiophanate-methyl (13 occurrences) and thiacloprid (10 occurrences), suggested a focus on fungal disease and insect pest management in these fruit crops.

The dominance of China (twenty-four occurrences) and pomelo (twenty-three occurrences), alongside with clothianidin (two occurrences) in Cluster 10 (Supplementary Materials S2—Table S2 and Figure S10), evidenced a targeted pesticide application pattern primarily linked to insect control in citrus production. This aligned with previous clusters where insecticide usage was emphasized in high-value fruit production such as Cluster 8 (lemons and pomegranates) and Cluster 9 (grapefruit and pears).

Cluster 11 (Supplementary Materials S2—Table S2 and Figure S11) highlighted a structured association between Brazilian agricultural exports (mainly watermelon) and the neonicotinoid insecticide imidacloprid. The presence of this in Brazil (two occurrences) and of watermelon (three occurrences), along with imidacloprid (fifteen occurrences), indicated a targeted pesticide application pattern primarily linked to insect control in watermelon production.

A limited but structured association between South African agricultural exports and pumpkin production was revealed by Cluster 12 (Supplementary Materials S2—Table S2 and Figure S12). The dominance of South Africa (thirty-eight occurrences) and the minor presence of pumpkin (one occurrence) implied a trade-based cluster rather than a strong pesticide-related association. This outcome agreed with previous research evidencing that clustering patterns in pesticide residue data are frequently driven by trade flows and supply chain dynamics versus only by chemical usage similarities. For example, it had been demonstrated that import origin significantly influences residue profiles due to centralized procurement and distribution networks [63]. Similarly, it had been reported that the multivariate analysis of pesticide residues in imported produce reflects market sourcing patterns, underscoring the role of trade in shaping contamination profiles [64]. Thus, the evidenced clustering reflected the predominance of South African imports within the studied dataset rather than specific pesticide application practices.

Expanding on the widespread presence of pesticide residues, in a study, the photodegradation and leaching behavior of 17 common pesticides was investigated in greenhouse soil, evidencing the presence of acetamiprid, carbendazim, metalaxyl, boscalid, and pyraclostrobin [65]. The results indicated that degradation rates were generally higher in light than in darkness, with significant leaching potential and a high potential risk of groundwater pollution for several pesticides such as chlorantraniliprole and metalaxyl. Additionally, boscalid and metalaxyl residues were detected in sediment and passive samples from Lake Ziway (Ethiopia), indicating significant influence from local agricultural activities, mostly linked to floriculture and small-scale vegetable farming. The findings suggested a historical presence of p,p’DDT (p,p′-dichlorodiphenyltrichloroethane) contamination while recent pesticide use trends showed a decrease in legacy pesticide usage, prompting the need for the ongoing monitoring of pesticide residues in the lake [66]. Consistent with these findings, a year-round study on pesticide contamination in public sites near intensively managed agricultural areas in South Tyrol (Italy) was conducted, evidencing that 96% of sampled sites were contaminated with at least one pesticide residue and 79% had multiple residues [67]. The highest concentration of pesticides was observed in spring, with some exceeding European maximum residue levels for food [52].

However, it is important to mention that some studies challenged regulatory reliance on active principle toxicity alone, emphasizing that adjuvants in formulations may drastically elevate human cell toxicity [68]. In order to reduce the impact of pesticides and pesticide residues, several studies underscored the importance of evaluating pesticide interactions, application timing, and concentration in optimizing pesticide compatibility on resistant strains to optimize pest control efficacy [69,70].

The cluster analysis revealed patterns that suggested both trade practices and pesticide usage trends across different regions and commodities. This interpretation aligned with earlier studies that had employed similar multivariate techniques to map pesticide residue distributions. For instance, a study evidenced the usage of high-resolution analytical methods in distinguishing residue trends across fruit and vegetable types, highlighting trade and agricultural practices as key contributors to clustering outcomes [64]. Moreover, a study identified the country of origin as a significant factor in shaping residue profiles in imported apples, emphasizing disparities in regulatory compliance and pesticide authorization among exporting nations [63]. The consistency between the results comprised within this study and the prior findings supports the robustness of the observed clusters and suggests that multivariate residue profiling may highlight underlying structures in global pesticide usage and supply chain behaviors. Thus, the identified clusters not only reflected analytical groupings but also echoed systemic patterns documented in international pesticide surveillance efforts.

In this context, the clustering analysis provided a systematic alignment between pesticide application patterns, crop-specific vulnerabilities, and international trade dynamics, with fungicides (e.g., pyraclostrobin, imazalil) and insecticides (e.g., methoxyfenozide, clothianidin) dominating distinct agricultural sectors. Regional agronomic practices, climatic conditions, and regulatory compliance with EU safety standards emerged as critical determinants of residue detection frequencies. While regulatory assessments (EPA, EFSA) indicate managed risks for most pesticides, persistent environmental concerns, such as groundwater contamination, endocrine disruption potential, and resistance development, necessitate enhanced surveillance and research into sustainable alternatives. These findings advocate for harmonized global monitoring frameworks to mitigate long-term ecological and public health impacts while ensuring trade-driven food security.

The originality of this research derived from the data-driven surveillance methodology, combining quantitative LC-MS/MS analysis with statistical tools such as ANOVA, clustering algorithms, and interactive visualizations. The aim was to reveal underlying pathways in pesticide residue contamination. With 620 fruit and vegetable analyzed samples and 74 screened pesticide compounds, this study may be considered one of the most comprehensive food safety assessments conducted in Romania up to date. Furthermore, the identification of region-specific and product-specific residue patterns, in particular the variations identified between EU and non-EU imports, may provide insight to the existing literature. While previous European monitoring initiatives, such as those by the EFSA, offered broad statistical summaries, the present investigation has delivered a more detailed, market-specific approach that can increase awareness regarding targeted regulatory actions and may increase public health risk evaluation.

## 4. Conclusions

This study provided an extensive overview of pesticide residue patterns in fruits and vegetables available on the Romanian market while focusing on the differentiation between EU and non-EU imports. A total of 620 samples were analyzed using LC-MS/MS and 74 pesticides were identified and quantified. The data revealed substantial variability in residue concentrations depending on the product type and geographic origin. Of particular interest, boscalid, azoxystrobin, and acetamiprid were among the most frequently detected compounds, particularly in strawberries, apples, and tomatoes. The results evidenced that most samples complied with EU MRLs; however, a subset approached or exceeded safety thresholds, mainly among imported products. For example, bupirimate levels in nectarines reached 0.039 ± 0.019 mg kg^−1^ (MRL of 0.3 mg kg^−1^) while difenoconazole in cherry tomatoes and broccoli reached 0.693 ± 0.346 mg kg^−1^ and 0.349 ± 0.174 mg kg^−1^, respectively, both below but close to their MRLs of 6 mg kg^−1^ and 1 mg kg^−1^ [33]. Azoxystrobin, acetamiprid, and boscalid were frequently found in concentrations above 0.2 mg kg^−1^ in imported fruits such as bananas, lemons, and strawberries. Moreover, imazalil residues in citrus fruits such as grapefruits and lemons ranged from 0.058 to 3.855 mg kg^−1^ (MRL of 4 mg kg^−1^), exceeding the stricter limit of 0.01 mg kg^−1^ applied to bananas [12]. These situations, mainly linked to non-EU imports (e.g., Egypt, Turkey, India), underlined the variable regulatory enforcement and differing pre-harvest practices in exporting countries. Despite the identified variations and occasional residues almost at regulatory limits, it is important to highlight that all detected pesticide levels remained below the established maximum residue limits (MRLs), confirming general compliance with EU safety requirements, even among non-EU imports from countries such as Turkey, Moldova, and Egypt.

Multivariate statistical tools, including ANOVA and K-means clustering, revealed significant trends and regional associations in pesticide usage. For instance, high concentrations of imazalil and pyrimethanil in citrus from Egypt and the frequent detection of acetamiprid in Greek and Romanian fruits evidenced specific trade-driven residue patterns. Clustering also highlighted phytosanitary practices tailored to pest pressure, crop type, and export market requirements. The co-occurrence of multiple residues, such as triazoles and neonicotinoids, underscored the importance of cumulative risk assessments, aligning with the EFSA’s PRIMo model recommendations [36].

Furthermore, the toxicological profiles of commonly detected pesticides such as acetamiprid [48,49,50,51], boscalid [52,53,54,55,56], methoxyfenozide [58], and hexythiazox [57] suggested potential risks not only to human health but also to pollinators, aquatic life, and ecosystems. The findings were consistent with the literature, evidencing the necessity for a broader understanding of pesticide formulation toxicity beyond active ingredients alone [68].

## 5. Future Perspectives

Although this study provided a comprehensive assessment of pesticide residues in fruits and vegetables on the Romanian market, various areas will demand further attention to increase food safety, environmental protection, and public health. The findings have significant implications for agricultural practices, trade policies, risk assessment, but also for consumer protection frameworks.

Although most samples complied with EU maximum residue limits (MRLs), the frequent detection of multiple residues, mainly in imported products, highlighted the necessity for policy evolution. EU regulations assess single compounds, but this investigation reinforced the relevance of cumulative exposure risk models, such as the EFSA’s PRIMo [39]. Regulatory authorities should update monitoring protocols to integrate multi-residue risk assessment and consider restricted import checks on high-risk commodities from non-EU countries, using digital flagging systems based on origin-residue profiles.

Current regulations assess only active ingredients, but adjuvants and synergists in commercial formulations may potence toxicity [68]. This deficiency requires full-formulation toxicity evaluation, mainly for persistent residues such as boscalid, acetamiprid, and methoxyfenozide [48,49,50,51,52,53,54,55,56,57,58]. Risk assessors and food safety agencies should require formulation-based toxicology surveys for new pesticide authorizations and reevaluate older formulations using updated chronic toxicity and mixture exposure criteria.

This research revealed geographical signatures in pesticide residues linked to export practices. Temporal and spatial surveillance may uncover usage tendencies and evidence systemic challenges in supply chains. Food safety regulators and importers should adopt seasonal monitoring plans and blockchain-based traceability systems to track pesticide use from “farm to shelf”, especially in high-volume export crops.

Many of the frequently detected pesticides (e.g., boscalid, hexythiazox) are persistent and ecotoxic [52,53,54,55,56,57]. This requires environmental co-monitoring alongside food testing to safeguard pollinators, aquatic ecosystems, and biodiversity. Environmental organisms should implement coordinated pesticide monitoring initiatives in agricultural areas, combining water, soil, and biota sampling to detect off-target contamination and guide buffer zone policy.

The use of Python, ANOVA, and clustering protocols enabled pattern discovery in large datasets. National officials and laboratories should combine automated statistical pipelines (e.g., outlier detection, clustering) into routine residue monitoring protocols to evidence non-compliance risks earlier and more efficiently.

Since main residue issues were more preponderant in non-EU imports, international harmonization is crucial. Exporting countries should align pesticide practices with EU regulations. Importers and authorities may request pre-export testing certificates and residue compliance history before market entry.

The cluster technique identified region-specific crop protection strategies. Agricultural advisors and producers may use this data to adjust pesticide application, refine integrated pest management (IPM) adoption, and decrease unnecessary chemical inputs through precision agriculture tools.

Due to the multiple residues detected even in compliant samples, public engagement is required. NGOs (Non-Governmental Organizations), retailers, and governments must encourage consumer awareness campaigns on washing practices, origin-based risks, and the importance of choosing certified low-residue or organic products. Retailers should also introduce pesticide transparency labels.

Summarizing, this research provided a framework not only for scientific inquiry but also for practical actions by various stakeholders. Addressing the raised issues requires a holistic, cross-sectoral strategy involving regulators, producers, researchers, and consumers. By integrating scientific insights with actionable steps, the EU in general, and Romania in particular, can move toward a more resilient, transparent, and sustainable food safety framework.

## Figures and Tables

**Table 1:** Optimized parameters for the 74 pesticide residues monitored in horticultural products from Romania.

No.	Compound	Elemental Composition	Molar Mass	Retention Time (min)	LOQ (mg/kg)	Precision (RSDR %)	Recovery (%)
Extremely hazardous (Class Ia) technical-grade active ingredients in pesticides according to WHO [27]							
1	Aldicarb	C7H14N2O2S	190.3	8.08	0.01	6.7516	100.00
2	Oxamyl	C7H13N3O3S	219.3	4.94	0.01	5.2186	99.42
Highly hazardous (Class Ib) technical-grade active ingredients in pesticides according to WHO [27]							
3	3-Hydroxy-carbofuran	C12H15NO4	237.2	6.84	0.005	10.6947	104.17
4	Carbofuran	C12H15NO3	221.3	9.4	0.005	7.7903	96.42
5	Fenamiphos	C13H22NO3PS	303.4	13.12	0.01	7.2163	94.75
6	Formetanate	C11H15N3O2	221.2	4.23	0.01	7.5405	98.50
7	Methiocarb	C11H15NO2S	225.3	11.84	0.01	8.187	98.92
8	Methomyl	C5H10N2O2S	162.2	5.11	0.01	11.3992	97.92
9	Monocrotophos	C7H14NO5P	223.2	5.79	0.01	5.9667	98.08
10	Omethoate	C5H12NO4PS	213.2	3.48	0.01	4.0786	87.80
11	Oxydemeton-methyl	C6H15O4PS2	246.3	5.43	0.01	8.8753	88.00
Moderately hazardous (Class II) technical-grade active ingredients in pesticides according to WHO [27]							
12	Acephate	C4H10NO3PS	183.2	2.66	0.01	7.3129	84.42
13	Acetamiprid	C10H11ClN4	222.7	7.59	0.01	10.692	98.83
14	Carbaryl	C12H11NO2	201.2	9.89	0.01	5.5741	96.75
15	Clothianidin	C6N5H8SO2Cl	249.6	6.52	0.01	6.4352	94.42
16	Cymoxanil	C7H10N4O3	198.2	7.16	0.01	7.8863	99.25
17	Cyproconazole (sum of isomers)	C15H18ClN3O	291.8	12.42	0.01	17.1248	94.08
18	Difenoconazole	C19H17Cl2N3O3	406.3	14.3	0.01	11.9048	89.92
19	Dimethoate	C5H12NO3PS2	229.3	6.55	0.01	6.5329	92.58
20	Fenpyroximate	C24H27N3O4	421.49	421.49	0.01	10.4407	98.42
21	Flutriafol	C16H13F2N3O	301.3	10.65	0.01	6.8064	93.50
22	Imazalil	C14H14Cl2N2O	297.2	10.84	0.01	14.5156	94.33
23	Imidacloprid	C9H10ClN5O2	255.7	6.82	0.01	4.8454	96.25
24	Indoxacarb	C22H17ClF3N3O7	527.84	14.15	0.01	10.6131	104.25
25	Isoprothiolane	C12H18O4S2	290.4	12.43	0.01	11.2825	99.92
26	Metalaxyl	C15H21NO4	279.3	11.19	0.01	14.3713	97.33
27	Oxadixyl	C14H18N2O4	278.4	9.13	0.01	12.5	96.08
28	Paclobutrazol	C15H20ClN3O	293.8	12.02	0.01	8.5577	100.92
29	Pirimicarb	C11H18N4O2	238.3	8.75	0.01	8.6874	96.25
30	Tebufenpyrad	C18H24ClN3O	333.9	14.76	0.01	6.1509	100.00
31	Thiacloprid	C10H9ClN4S	252.7	8.35	0.01	6.2416	92.50
32	Thiamethoxam	C8H10ClN5O3S	291.7	5.59	0.01	5.3174	91.17
33	Thiodicarb	C10H18N4O4S3	354.5	11.27	0.01	7.8327	102.67
34	Triadimefon	C14H16ClN3O2	293.8	12.41	0.01	11.5317	95.75
Slightly hazardous (Class III) technical-grade active ingredients in pesticides according to WHO [27]							
35	Bupirimate	C13H24N4O3S	316.4	12.85	0.01	12.8778	103.92
36	Buprofezin	C16H23N3OS	305.4	14.7	0.01	7.5534	102.83
37	Clofentezine	C14H8Cl2N4	303.1	13.88	0.01	7.4513	102.50
38	Cyprodinil	C14H15N3	225.3	12.88	0.01	13.7595	93.17
39	Diflubenzuron	C14H9ClF2N2O2	310.7	12.84	0.01	13.0973	103.33
40	Dimethomorph (sum of isomers)	C21H22ClNO4	387.9	12.39	0.01	10.0078	102.92
41	Fenamidone	C17H17N3OS	311.4	11.79	0.01	4.0587	110.42
42	Flufenoxuron	C21H11ClF6N2O3	488.8	15.26	0.01	7.5466	97.75
43	Hexaconazole	C14H17Cl2N3O	314.2	13.58	0.01	10.3533	95.25
44	Linuron	C9H10Cl2N2O2	249.1	11.57	0.01	10.8634	98.50
45	Lufenurone (sum of isomers)	C17H8Cl2F8N2O3	511.15	15.05	0.01	4.0473	104.58
46	Penconazole	C13H15Cl2N3	284.2	13.45	0.01	9.3743	99.50
47	Propargite	C19H26O4S	350.5	15.37	0.01	7.9044	102.58
48	Prothioconazole (sum of isomers)	C14H15Cl2N3OS	344.3	13.71	0.01	8.2491	105.33
49	Pyrimethanil	C12H13N3	199.3	11.06	0.01	7.3721	91.75
50	Tau-fluvalinate	C26H22ClF3N2O3	502.9	16.07	0.01	5.796	94.50
51	Terbuthylazine	C9H16ClN5	229.7	12.03	0.01	6.1566	95.75
Technical-grade active ingredients of pesticides unlikely to present acute hazard in normal use according to WHO [27]							
52	Azoxystrobin	C22H17N3O5	403.4	12.17	0.01	9.874	97.67
53	Boscalid	C18H12Cl2N2O	343.2	12.05	0.01	8.9444	95.25
54	Carbendazim	C9H9N3O2	191.1	5.48	0.01	13.9739	89.67
55	Diethofencarb	C14H21NO4	267.3	11.64	0.01	12.4197	104.75
56	Etofenprox	C25H28O3	376.5	16.5	0.01	5.7503	103.25
57	Fenhexamid	C14H17Cl2NO2	302.2	12.52	0.01	18.0704	78,00
58	Fenoxycarb	C17H19NO4	301.4	13.09	0.01	9.0561	97.00
59	Hexythiazox	C17H21ClN2O2S	352.9	14.98	0.01	7.0232	99.50
60	Iprovalicarb	C18H28N2O3	320.4	12.51	0.01	10.1103	102.75
61	Mandipropamid	C23H22ClNO4	411.9	12.19	0.01	5.1845	92.17
62	Mepanipyrim	C16H8Cl2FN5O	223.3	12.64	0.01	12.8706	98.94
63	Pyriproxyfen	C20H19NO3	321.4	14.93	0.01	5.1281	102.42
64	Tebufenozide	C22H28N2O2	352.5	13.16	0.01	8.6746	102.08
65	Thiophanate-methyl	C12H14N4O4S2	342.3	9.21	0.01	5.6517	97.58
66	Trifloxystrobin	C20H19F3N2O4	408.4	14.28	0.01	8.6409	101.00
67	Triflumuron	C15H10ClF3N2O3	358.7	13.64	0.01	8.0693	94.53
Not classified by WHO [27]							
68	Aldicarb sulfone	C7H14N2O4S	222.3	8.07	0.01	4.8834	100.67
69	Aldicarb sulfoxide	C7H14N2O3S	206.3	4.22	0.01	6.3513	100.67
70	Epoxiconazole	C17H13ClFN3O	329.8	13.03	0.01	13.0611	98.92
71	Fluquinconazole	C16H8Cl2FN5O	376.2	12.59	0.01	15.0897	98.67
72	Methiocarb-Sulfoxide	C11H15NO3S	241.3	7.39	0.01	9.2582	93.33
73	Methoxyfenozide	C22H28N2O3	368.5	12.45	0.01	9.0269	102.58
74	Pyraclostrobin	C19H18ClN3O4	387.8	13.84	0.01	8.0628	91.00

Note: Linearity was verified in the range of 5–200 µg/L, with correlation coefficients (R^2^) = 0.99 for all compounds. Fortification levels were set at 0.01 mg/kg for recovery assessment.

**Table 2:** Assessment of the influence of various variables on pesticide residues in fruits and vegetables via analysis of variance.

Index	Sum sq	df	F	PR(>F)
Vegetables				
C(Continent)	0.00024	2	0.012	0.911
C(Pesticide)	0.59878	25	2.511	0.061
C(Continent):C(Pesticide)	0.40530	50	0.850	0.712
Residual	1.37328	144	–	–
Fruits				
C(Continent)	0.35979	5	0.236	1.000
C(Pesticide)	0.00013	41	<0.001	0.789
C(Continent):C(Pesticide)	74.52770	205	1.194	0.105
Residual	301.32443	990	–	–

**Table 3:** Summary of the pesticides in fruits and vegetables and counted outliers.

Pesticide	Outlier_Count in Fruits	Outlier_Count in Vegetables
Acetamiprid	12	4
Azoxystrobin	13	-
Boscalid	6	3
Buprofezin	1	-
Carbendazim	2	-
Cyprodinil	2	-
Difenoconazole	5	2
Dimethoate	1	-
Dimethomorph (sum of isomers)	1	-
Hexythiazox	1	-
Imazalil	2	-
Imidacloprid	2	1
Propiconazole (sum of isomers)	1	-
Pyraclostrobin	3	1
Pyrimethanil	8	1
Pyriproxyfen	6	1
Tebufenpyrad	1	-
Thiacloprid	1	-
Thiophanate-Methyl	1	-
Trifloxystrobin	2	-
Methoxyfenozide	-	1
Indoxacarb	-	1

## Data Availability

The original contributions presented in this study are included in the article/Supplementary Material. Further inquiries can be directed to the corresponding author(s).

## Supplementary Materials

The following supporting information can be downloaded at: https://www.mdpi.com/article/10.3390/jox15040104/s1, Supplementary Materials S1: List of analysed samples and screened pesticides; Table S1: Clusters 1–6: Terms and occurrences; Table S2: Clusters 7–12: Terms and occurrences; Figure S1: Cluster 1 connections; Figure S2: Cluster 2 connections; Figure S3: Cluster 3 connections; Figure S4: Cluster 4 connections; Figure S5: Cluster 5 connections; Figure S6: Cluster 6 connections; Figure S7: Cluster 7 connections; Figure S8: Cluster 8 connections; Figure S9: Cluster 9 connections; Figure S10: Cluster 10 connections; Figure S11: Cluster 11 connections; Figure S12: Cluster 12 connections.

## Abbreviations

The following abbreviations were used in this manuscript:

QuEChERS	Quick, Easy, Cheap, Effective, Rugged, Safe
LC-MS/MS	liquid chromatography–tandem mass spectrometry
MRLs	maximum residue limits
EU	European Union
CODEX	Codex Alimentarius Commission
GCB	graphitized carbon black
FAO	Food and Agriculture Organization of the United Nations
WHO	World Health Organization
UNEP	United Nations Environment Programme
SDG	Sustainable Development Goal
HPLC	high-performance liquid chromatography
ESI	electrospray ionization
TPP	triphenyl phosphate
PSA	primary secondary amine
SPE	solid-phase extraction
DSPE	dispersive solid-phase extraction
EDA	Exploratory Data Analysis
ANOVA	analysis of variance
Sum_sq	sum of squares
SDHIs	succinate dehydrogenase inhibitors
ATP	adenosine triphosphate
p,p’DDT	p,p′-dichlorodiphenyltrichloroethane
EFSA	European Food Safety Authority
ROS	reactive oxygen species
EPA	U.S. Environmental Protection Agency
LC50	median lethal concentration
RNA	ribonucleic acid
IPM	integrated pest management
RfD	reference dose
MOEs	margins of exposure
