# Supplementary material for: Pesticide Surveillance in Fruits and Vegetables from Romanian Supply: A Data-Driven Approach
Source: J Xenobiot. 2025 Jul 2;15(4):104. doi: 10.3390/jox15040104 (PMC12285958; doi:10.3390/jox15040104)
Supplement: Supplementary file 1 [file jox-15-00104-s001.zip › Supplementary Material_S2.pdf]

Supplementary material:

Pesticide Surveillance in Fruits and Vegetables from Romanian Supply: A Data-Driven Approach

Diana Ionela Popescu (Stegarus), Ana-Maria Nasture, Violeta-Carolina Niculescu, Corina Mihaela Oprita (Cioara) and Nicoleta Anca Șuțan (Ionescu)

Table S1. Clusters 1-6 terms and occurrences

<table>
<tr><th colspan="2">Cluster 1</th><th colspan="2">Cluster 2</th><th colspan="2">Cluster 3</th><th colspan="2">Cluster 4</th><th colspan="2">Cluster 5</th><th>Cluster 6</th><th></th></tr>
<tr><th>Pescicide</th><th>Occurrence</th><th>Pescicide</th><th>Occurrence</th><th>Pescicide</th><th>Occurrence</th><th>Pescicide</th><th>Occurrence</th><th>Pescicide</th><th>Occurrence</th><th>Pescicide</th><th>Occurrence</th></tr>
<tr><td>pyraclostrobin</td><td>73</td><td>grape</td><td>41</td><td>Turkey</td><td>201</td><td>apple</td><td>42</td><td>azoxystrobin</td><td>137</td><td>imazalil</td><td>245</td></tr>
<tr><td>boscalid</td><td>52</td><td>tomato</td><td>27</td><td>cucumber</td><td>18</td><td>difenoconazole</td><td>40</td><td>pyriproxyfen</td><td>85</td><td>pyrimethanil</td><td>207</td></tr>
<tr><td>acetamiprid</td><td>47</td><td>hexythiazox</td><td>17</td><td>Spain</td><td>13</td><td>Moldova</td><td>32</td><td>banana</td><td>78</td><td>orange</td><td>84</td></tr>
<tr><td>cyprodinil</td><td rowspan="2">38</td><td>methoxyfenozide</td><td>15</td><td>Albania</td><td>12</td><td>Poland</td><td>20</td><td>Ecuador</td><td>33</td><td>Egypt</td><td>70</td></tr>
<tr><td>Greece</td><td>netherland</td><td>13</td><td>metalaxyl</td><td>11</td><td>plum</td><td rowspan="2">5</td><td>Costa Rica</td><td>25</td><td>mandarin</td><td>37</td></tr>
<tr><td>pepper</td><td>31</td><td>indoxacarb</td><td>12</td><td>carrot</td><td rowspan="2">6</td><td>Italy</td><td>Columbia</td><td>14</td><td>tau-fluvalinate</td><td>14</td></tr>
<tr><td>Romania</td><td>13</td><td>fenhexamid</td><td>9</td><td>clementine</td><td>Macedonia</td><td>4</td><td>Guatemala</td><td>2</td><td>Guatemala</td><td>12</td></tr>
<tr><td>penconazol</td><td>10</td><td>mandipropamid</td><td>4</td><td>hungary</td><td>4</td><td>diflubenzuron</td><td>3</td><td>Cote d’ivoire</td><td rowspan="3">1</td><td>fenpyroximate</td><td>6</td></tr>
<tr><td>apricot</td><td>9</td><td>flutriafol</td><td>3</td><td>pea</td><td>2</td><td>propargite</td><td>2</td><td>French Antilles</td><td>Zimbabwe</td><td>2</td></tr>
<tr><td>strawberry</td><td>8</td><td>cabbage</td><td rowspan="4">1</td><td>linuron</td><td rowspan="2">1</td><td>leek</td><td rowspan="3">1</td><td>Mexico</td><td>garlic</td><td>1</td></tr>
<tr><td>Serbia</td><td>6</td><td>celery</td><td>zucchini</td><td>fenoxycarb</td><td></td><td></td><td></td><td></td></tr>
<tr><td>peach</td><td>5</td><td>thiamethoxa</td><td></td><td></td><td>broccoli</td><td></td><td></td><td></td><td></td></tr>
<tr><td>nectarine</td><td rowspan="2">4</td><td>Tunisia</td><td></td><td></td><td></td><td></td><td></td><td></td><td></td><td></td></tr>
<tr><td>bupirimate</td><td></td><td></td><td></td><td></td><td></td><td></td><td></td><td></td><td></td><td></td></tr>
<tr><td>eggplant</td><td rowspan="3">2</td><td></td><td></td><td></td><td></td><td></td><td></td><td></td><td></td><td></td><td></td></tr>
<tr><td>etofenprox</td><td></td><td></td><td></td><td></td><td></td><td></td><td></td><td></td><td></td><td></td></tr>
<tr><td>trifloxystrobin</td><td></td><td></td><td></td><td></td><td></td><td></td><td></td><td></td><td></td><td></td></tr>
<tr><td>cherry</td><td rowspan="5">1</td><td></td><td></td><td></td><td></td><td></td><td></td><td></td><td></td><td></td><td></td></tr>
<tr><td>kiwi</td><td></td><td></td><td></td><td></td><td></td><td></td><td></td><td></td><td></td><td></td></tr>
<tr><td>lettuce</td><td></td><td></td><td></td><td></td><td></td><td></td><td></td><td></td><td></td><td></td></tr>
<tr><td>sour cherries</td><td></td><td></td><td></td><td></td><td></td><td></td><td></td><td></td><td></td><td></td></tr>
<tr><td>tebufenozide</td><td></td><td></td><td></td><td></td><td></td><td></td><td></td><td></td><td></td><td></td></tr>
</table>

Table S2. Clusters 7-12 terms and occurrences

Cluster 7		Cluster 8		Cluster 9		Cluster 10		Cluster 11		Cluster 12	
Pescicide	***Occurrence***	***Pescicide***	***Occurrence***	***Pescicide***	***Occurrence***	***Pescicide***	***Occurrence***	***Pescicide***	***Occurrence***	***Pescicide***	***Occurrence***
dimethomorph	49	lemon	84	grapefruit	47	China	24	Brazil	2	South Africa	38
lufenuron	33	pomegranate	11	Argentina	26	pomelo	23	watermelon	3	pumpkin	1
India	7	buprofezin	5	pear	21	clothianidin	2	imidacloprid	15		
Ukraine	3	dimethoate		thiophanate-methyl	13						
onion		Peru		thiacloprid	10						
cymoxanil	1	tebufenpyrad	4	quince	5						
Uruguay		pirimicarb		isoprothiolane	1						
		ometoate	2	Portugal							
		Israel	1								

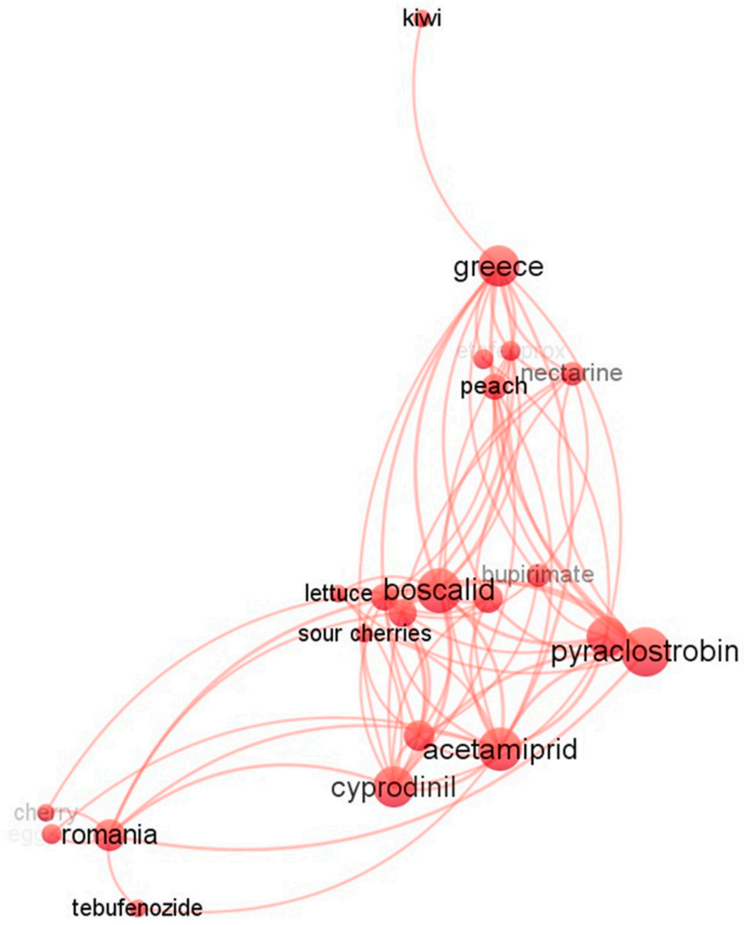

Figure S1. Cluster 1 connections

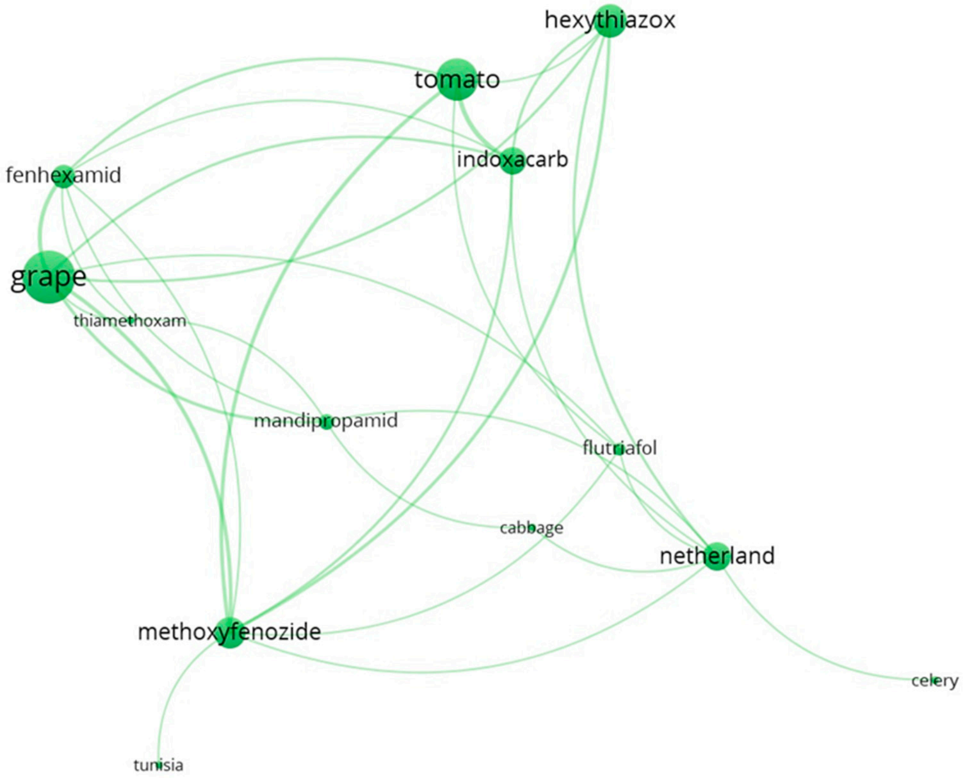

Figure S2. Cluster 2 connections

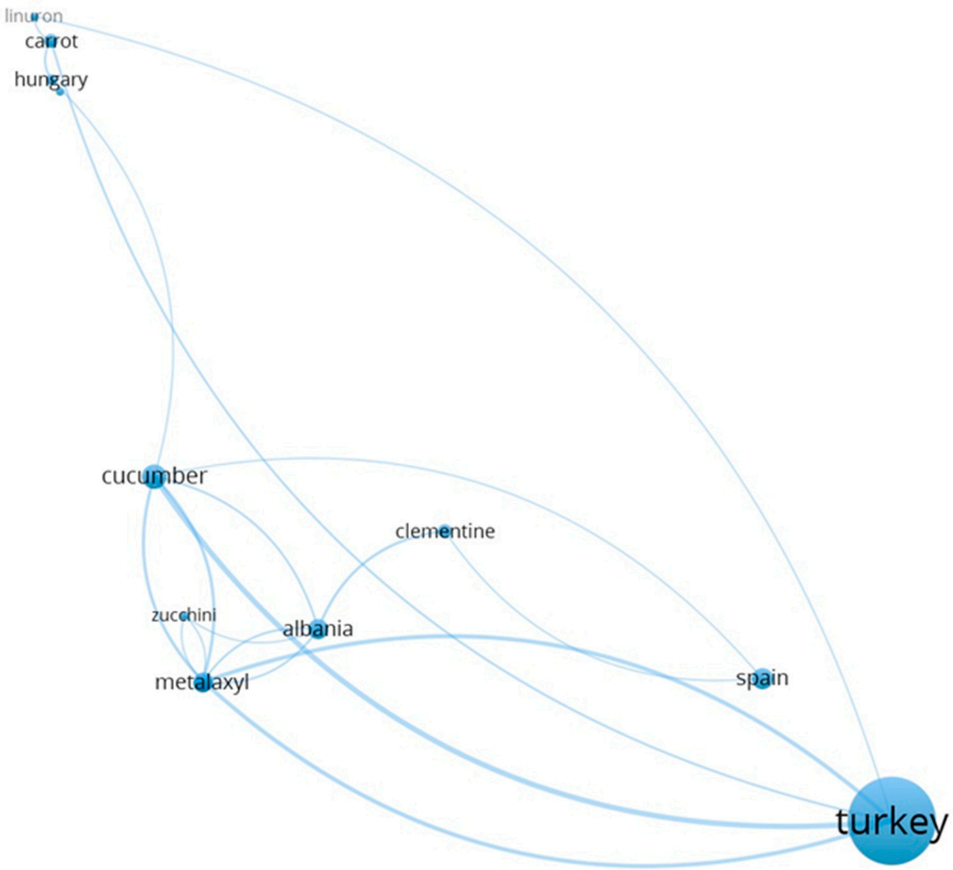

Figure S3. Cluster 3 connections

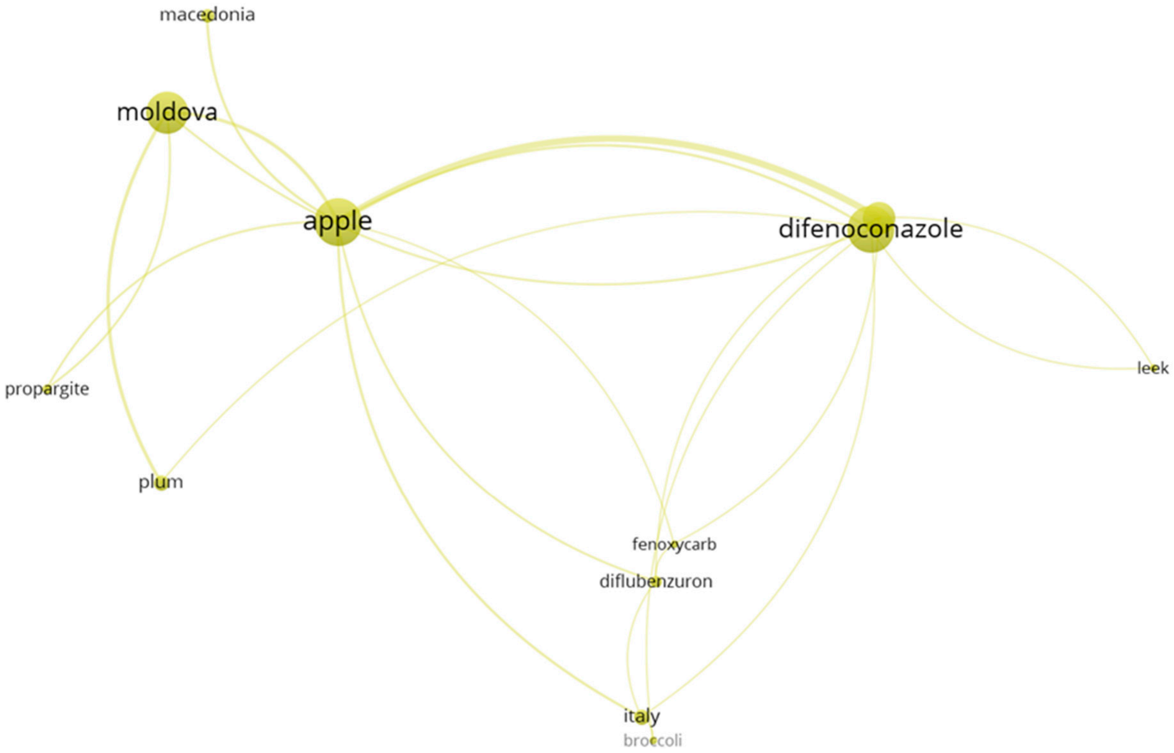

Figure S4. Cluster 4 connections

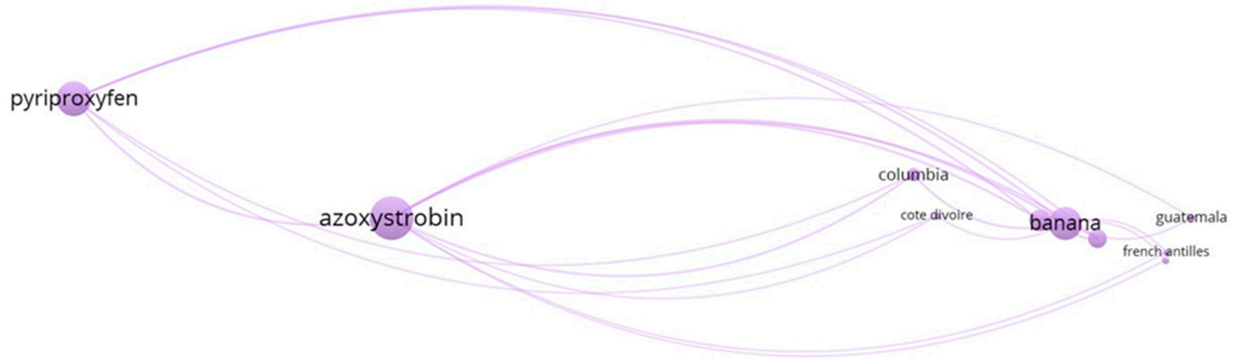

Figure S5. Cluster 5 connections

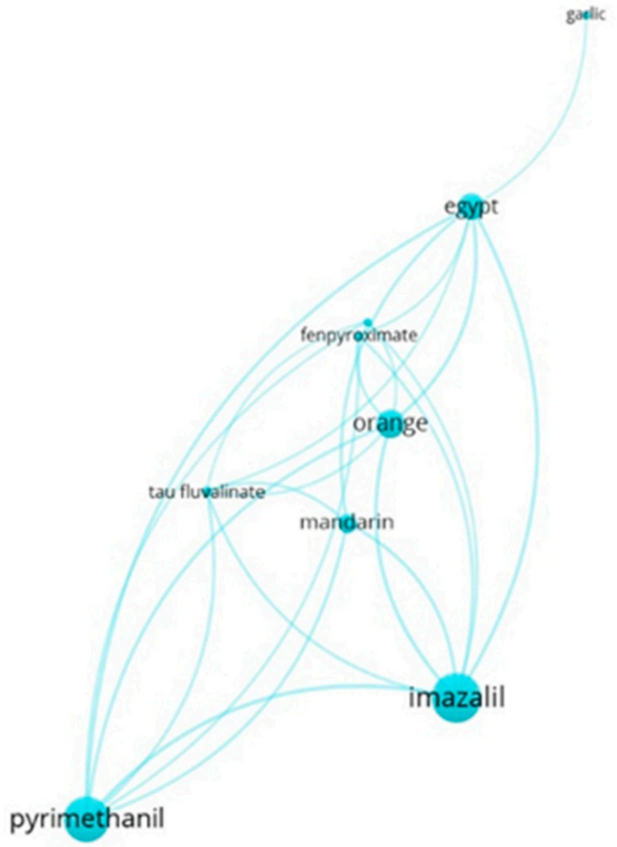

Figure S6. Cluster 6 connections

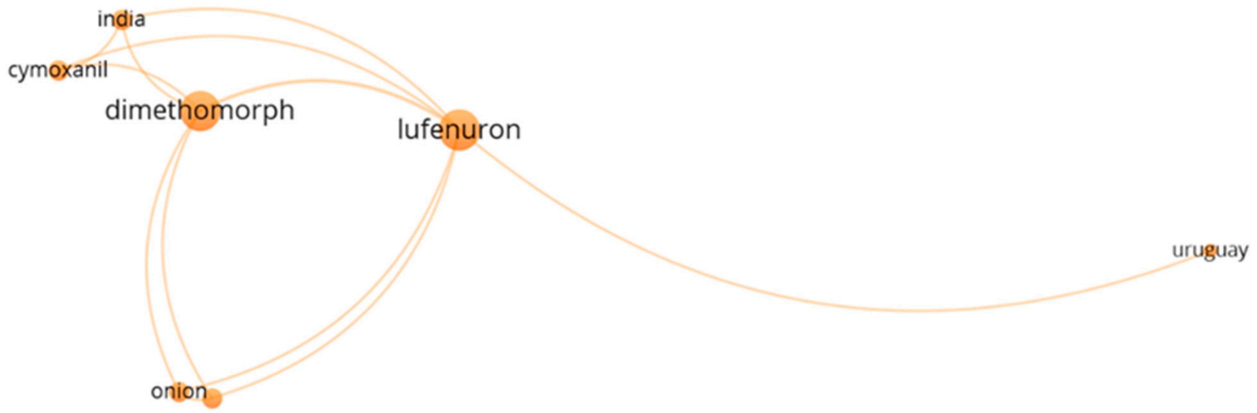

Figure S7. Cluster 7 connections

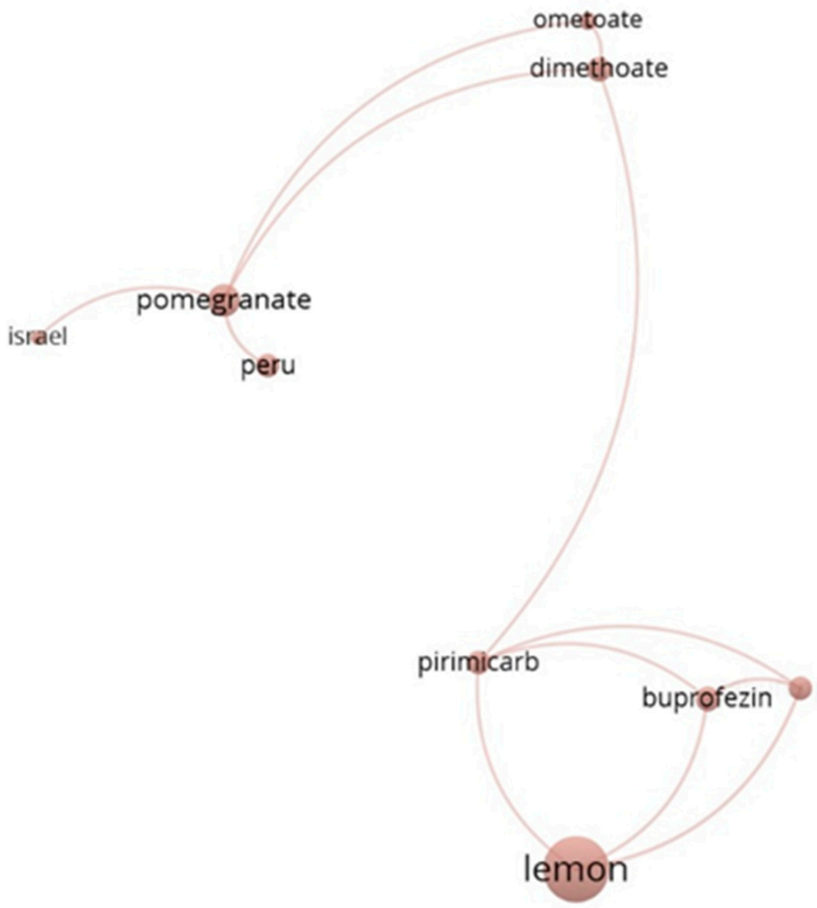

Figure S8. Cluster 8 connections

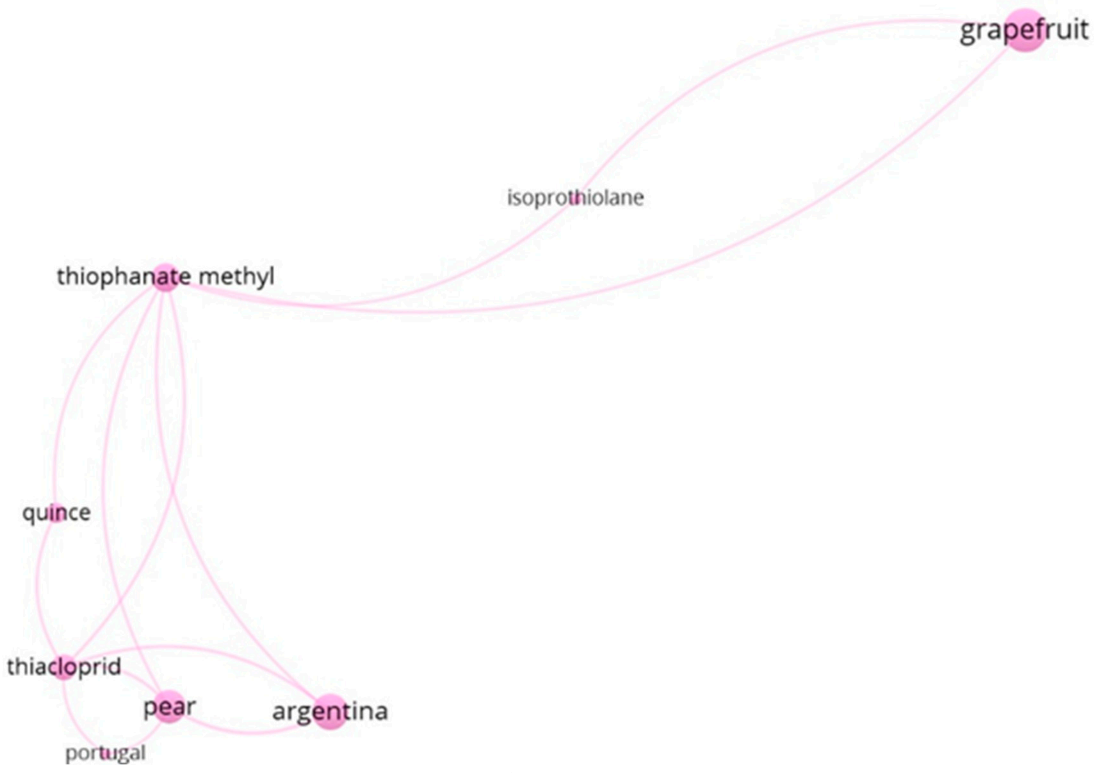

Figure S9. Cluster 9 connections

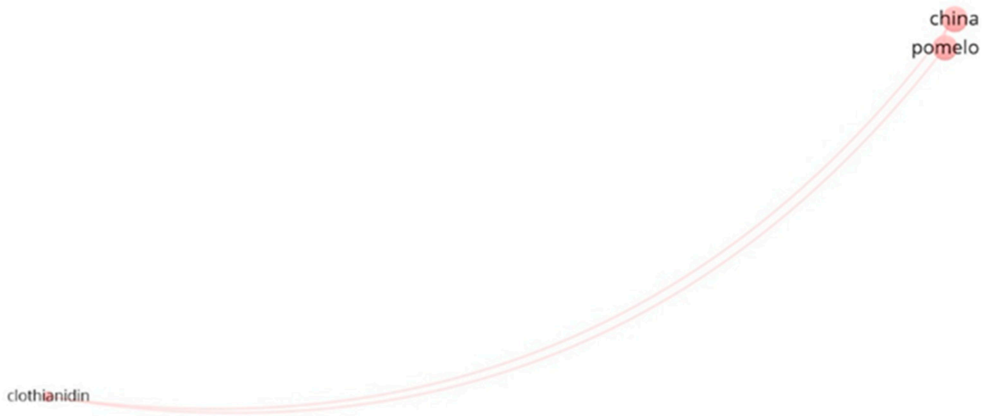

Figure S10. Cluster 10 connections

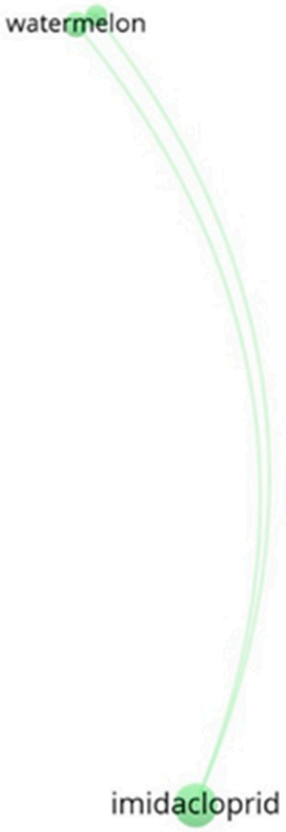

Figure S11. Cluster 11 connections

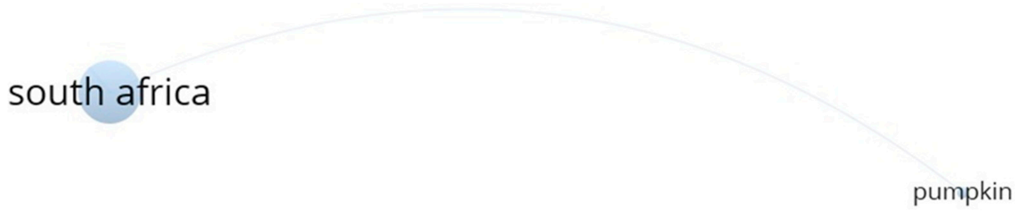

Figure S12. Cluster 12 connections
